# Chromosome-scale genome assembly of *Cucumis hystrix*—a wild species interspecifically cross-compatible with cultivated cucumber

**DOI:** 10.1038/s41438-021-00475-5

**Published:** 2021-03-01

**Authors:** Xiaodong Qin, Zhonghua Zhang, Qunfeng Lou, Lei Xia, Ji Li, Mengxue Li, Junguo Zhou, Xiaokun Zhao, Yuanchao Xu, Qing Li, Shuqiong Yang, Xiaqing Yu, Chunyan Cheng, Sanwen Huang, Jinfeng Chen

**Affiliations:** 1 State Key Laboratory of Crop Genetics and Germplasm Enhancement, Nanjing Agricultural University, 210095 Nanjing, China; 2 College of Horticulture, Qingdao Agricultural University, 266109 Qingdao, China; 3 Institute of Vegetables and Flowers, Chinese Academy of Agricultural Sciences, 100081 Beijing, China; 4 College of Horticulture and Landscape, Henan Institute of Science and Technology, 453003 Xinxiang, China; 5 Agricultural Genomics Institute, Chinese Academy of Agricultural Sciences, 518120 Shenzhen, China

**Keywords:** Genome, Evolution

## Abstract

*Cucumis hystrix* Chakr. (2n = 2x = 24) is a wild species that can hybridize with cultivated cucumber (*C. sativus* L., 2n = 2x = 14), a globally important vegetable crop. However, cucumber breeding is hindered by its narrow genetic base. Therefore, introgression from *C. hystrix* has been anticipated to bring a breakthrough in cucumber improvement. Here, we report the chromosome-scale assembly of *C. hystrix* genome (289 Mb). Scaffold N50 reached 14.1 Mb. Over 90% of the sequences were anchored onto 12 chromosomes. A total of 23,864 genes were annotated using a hybrid method. Further, we conducted a comprehensive comparative genomic analysis of cucumber, *C. hystrix*, and melon (*C. melo* L., 2n = 2x = 24). Whole-genome comparisons revealed that *C. hystrix* is phylogenetically closer to cucumber than to melon, providing a molecular basis for the success of its hybridization with cucumber. Moreover, expanded gene families of *C. hystrix* were significantly enriched in “defense response,” and *C. hystrix* harbored 104 nucleotide-binding site–encoding disease resistance gene analogs. Furthermore, 121 genes were positively selected, and 12 (9.9%) of these were involved in responses to biotic stimuli, which might explain the high disease resistance of *C. hystrix*. The alignment of whole *C. hystrix* genome with cucumber genome and self-alignment revealed 45,417 chromosome-specific sequences evenly distributed on *C. hystrix* chromosomes. Finally, we developed four cucumber–*C. hystrix* alien addition lines and identified the exact introgressed chromosome using molecular and cytological methods. The assembled *C. hystrix* genome can serve as a valuable resource for studies on *Cucumis* evolution and interspecific introgression breeding of cucumber.

## Introduction

**Fig. 1:**
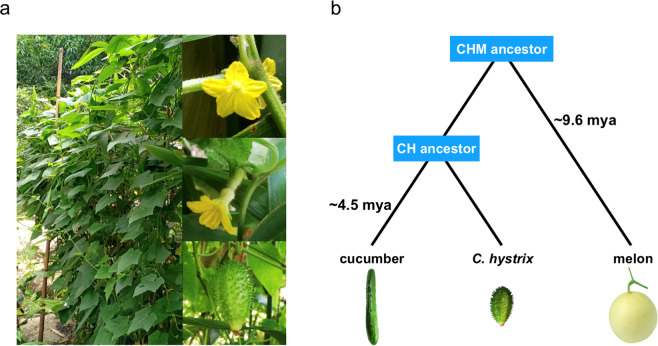
Species information of *Cucumis hystrix*. **a** Morphological characteristics of *C. hystrix*. (left, adult plant; top right, male flower; middle right, female flower; bottom right, fruit). **b** Evolutionary relationships of cucumber (C), *C. hystrix* (H), and melon (M) and their divergence time (mya, million years ago)

*Cucumis hystrix* Chakr. (2n = 2x = 24) is a wild perennial congener of cucumber (*C. sativus* L., 2n = 2x = 14) and melon (*C. melo* L., 2n = 2x = 24). It is a climber and grows in bushes on hills at ~1 km above the mean sea level, particularly along the streams where the sunlight is poor and humidity is high (Fig. 1a, left). It is geographically distributed in Southeast Asia, from South China to Myanmar, Thailand, Bangladesh, and Northeast India^1^. The fruit of *C. hystrix* has a cucumber-like and slightly sour taste (Fig. 1a, bottom right). The stem of an adult *C. hystrix* plant can gradually become semi-lignified and crack during development. Male and female flowers of *C. hystrix* (Fig. 1a, top and middle right, respectively) are almost identical to those of cucumber, but smaller. It can overwinter in the native regions.

*C. hystrix* has attracted much attention because of its cross-compatibility with cucumber^2^ as well as resistance to biotic stresses (e.g., root knot and downy mildew^3^) and tolerance of abiotic stresses (e.g., low sunlight^4^ and low temperature^5^). Cucumber is a valuable vegetable crop and widely consumed worldwide. However, the genetic base of cucumber has become increasingly narrow due to long-term and directed domestication, which is a hurdle in cucumber breeding^6^. Wild species possess abundant natural variations, which are absent in crops, and these variations can potentially enrich the gene pool of crops and further improve the desirable target traits^7–10^.

A new interspecific hybrid of *Cucumis* was successfully developed by doubling the chromosomes of the sterile F1 generation (2n = 2x = 19) of cucumber and *C. hystrix*, giving rise to the allotetraploid *Cucumis* *×* *hytivus* J.-F. Chen & J. H. Kirkbr. (*C. hytivus*, 2n = 4x = 38). Successful hybridization of cucumber and *C. hystrix* proved to be a cornerstone of cucumber interspecific breeding. Following this, a number of introgression lines were developed through recurrent backcrossing of this artificial allotetraploid to cucumber, and some of these lines exhibited substantially increased disease resistance^11^. Genetic assessment of *C. hytivus*-derived inbred backcross lines indicated that the genetic diversity of cucumber was broadened^12^.

Genome sequencing can identify abundant molecular markers with full coverage and high specificity and accuracy to trace the introgressed segments, which is crucial for interspecific introgression breeding. Therefore, high-quality genome assembly of *C. hystrix* is imperative to identify efficient interspecific hybrid materials and develop genetic resources for cucumber improvement.

Genomic data of flowering plants are rapidly accumulating^13^. The cucumber whole genome—the first genome of a vegetable crop—was compiled in 2009^14^, which heralded the dawn of the genomics-directed era of vegetable breeding. The genome of melon, another economically important *Cucumis* crop, was also compiled 3 years later^15^. The evolutionary relationships of the three *Cucumis* species are shown in Fig. 1b. A three-way comparison can be used to track the potential events driving speciation. Previous studies assessed the phylogenetic relationships of *Cucumis* species using selected molecular markers^16–19^, cytological methods^20,21^, and genetic linkage maps^22^. Nevertheless, these methods have limited power to reveal the phylogenetic relationships among species, and considering the complex factors, such as incomplete lineage sorting, interspecific hybridization-induced gene flow, and horizontal transfer, different data or computing methods may reveal diverse evolutionary history^23–26^. In this context, genome-scale comparative analysis can provide comprehensive and robust information for elucidating evolutionary events.

The genome of *C. hystrix* was preliminarily assembled in a previous study^27^, albeit with low coverage and continuity and without full annotation. This assembly is far from satisfactory, and the lack of a high-quality *C. hystrix* reference genome has indeed impeded the comparative genomic analyses of *Cucumis* species. To this end, the results of the present study provide an invaluable resource for uncovering the evolutionary events of *Cucumis* species and improving cucumber via interspecific hybridization.

## Results

### *C. hystrix* genome assembly and quality assessment

The estimated genome size, heterozygosity, and repeat content of the *C. hystrix* genome were 416 Mb, 0.78%, and 53.5%, respectively. We assembled the *C. hystrix* genome using a hybrid method with different datasets (Table S1). Supernova^28^ was used to assemble the 10× genomic data of the recommended size using default parameters. Contig N50 (minimum contig length representing half of the total length of the assembly) of the Supernova assembly was 108 kb, and its scaffold N50 (minimum scaffold length representing half of the total length of the assembly) was 7.6 Mb. We conducted further gap-filling, polishing, and scaffolding using self-corrected PacBio, pair-end, and mate-pair data. A general workflow of the assembly is presented in Fig. S1. We finally assembled 289 Mb sequences—approximately 80 Mb more than the previously published assembly^27^. The contig N50 was 221 kb, and the scaffold N50 was 14 Mb, with a 100- and 277-fold improvement, respectively. Moreover, 90.4% of the assembled scaffolds were anchored and 88.2% were oriented on 12 pseudochromosomes based on 416 markers in a linkage map developed in a previous study^27^. The overall scaffold anchoring statistics are summarized in Table S2, and the final assembly statistics are summarized in Table 1. The GC content was 33.12%, and the repeat sequences constituted 48.7% of the genome, with long terminal repeats being the most abundant (19.64%). Repeat statistics of the assembly are summarized in Table S3. We predicted 23,864 gene models using a hybrid method based on ab initio, homology alignment, and transcriptome sequencing of five tissues (root, stem, leaf, male flower, and ovary). The results of a simple comparison of genome assembly among the three *Cucumis* species (cucumber, *C. hystrix*, and melon) are summarized in Table S4. The genome size of *C. hystrix* was estimated to be larger than that of cucumber but smaller than that of melon, and the total size of the assembled sequences was in the same order.

**Table 1:** Statistics of *Cucumis hystrix* draft assembly

Statistics	Value
Total size of assembled contigs (bp)	289,989,644
Number of contigs (>100 bp)	6072
Largest contig (bp)	1,438,864
Contig N50 (bp)	220,950
Total size of assembled scaffolds (bp)	297,500,035
Number of scaffolds (>300 bp)	2284
Largest scaffold (bp)	20,059,872
Scaffold N50 (bp)	14,064,021
Sequences anchored on chromosomes (bp)	268,892,684 (90.4%)
Sequences oriented on chromosomes (bp)	262,424,878 (88.2%)

We evaluated the quality of the genome using various methods. There was acceptable consistency between the assembly and linkage groups (Fig. S2). We randomly selected a region of chromosome 2 and found that most of it was supported by considerable mate-pair reads (Fig. S3). Of the 1440 single-copy orthologous genes from BUSCO^29^, respectively, 1307 (90.7%) and 31 (2.2%) were assigned as complete and fragmented in the *C. hystrix* draft genome. A total of 1323 (91.9%) complete and 46 (3.2%) fragmented single-copy orthologous genes were detected in 23,864 putative proteins. The BUSCO results were comparable to those of several other published genome assemblies of Cucurbitaceae species (Table S5). All assessment results indicated that the *C. hystrix* genome assembly was of high quality.

### Similarities among cucumber, *C. hystrix*, and melon at the nucleotide and protein levels

We conducted comprehensive pairwise whole-genome alignments using the assembled genomes of cucumber, *C. hystrix*, and melon and annotated their proteomes. Specifically, 223.1 Mb (74.9%) sequences of *C. hystrix* were aligned to 199.0 Mb (88.0%) sequences of cucumber, and 161.0 (54.1%) and 160.2 Mb (70.8%) sequences of *C. hystrix* and cucumber, respectively, showed one-to-one correspondence. Meanwhile, only 156.1 Mb (52.4%) sequences of *C. hystrix* could be aligned to 172.3 Mb (41.4%) sequences of melon using the same alignment parameters, with only 111.5 (37.5%) and 111.7 Mb (26.8%) sequences of *C. hystrix* and melon, respectively, showing one-to-one correspondence. Cucumber, *C. hystrix*, and melon genomes contained 25.3, 62.7, and 238.1 Mb species-specific (no hits for either of the other two species) sequences, respectively. The average identity of the aligned sequences was 91.55% between *C. hystrix* and cucumber, 89.29% between *C. hystrix* and melon, and 89.56% between cucumber and melon.

**Fig. 2:**
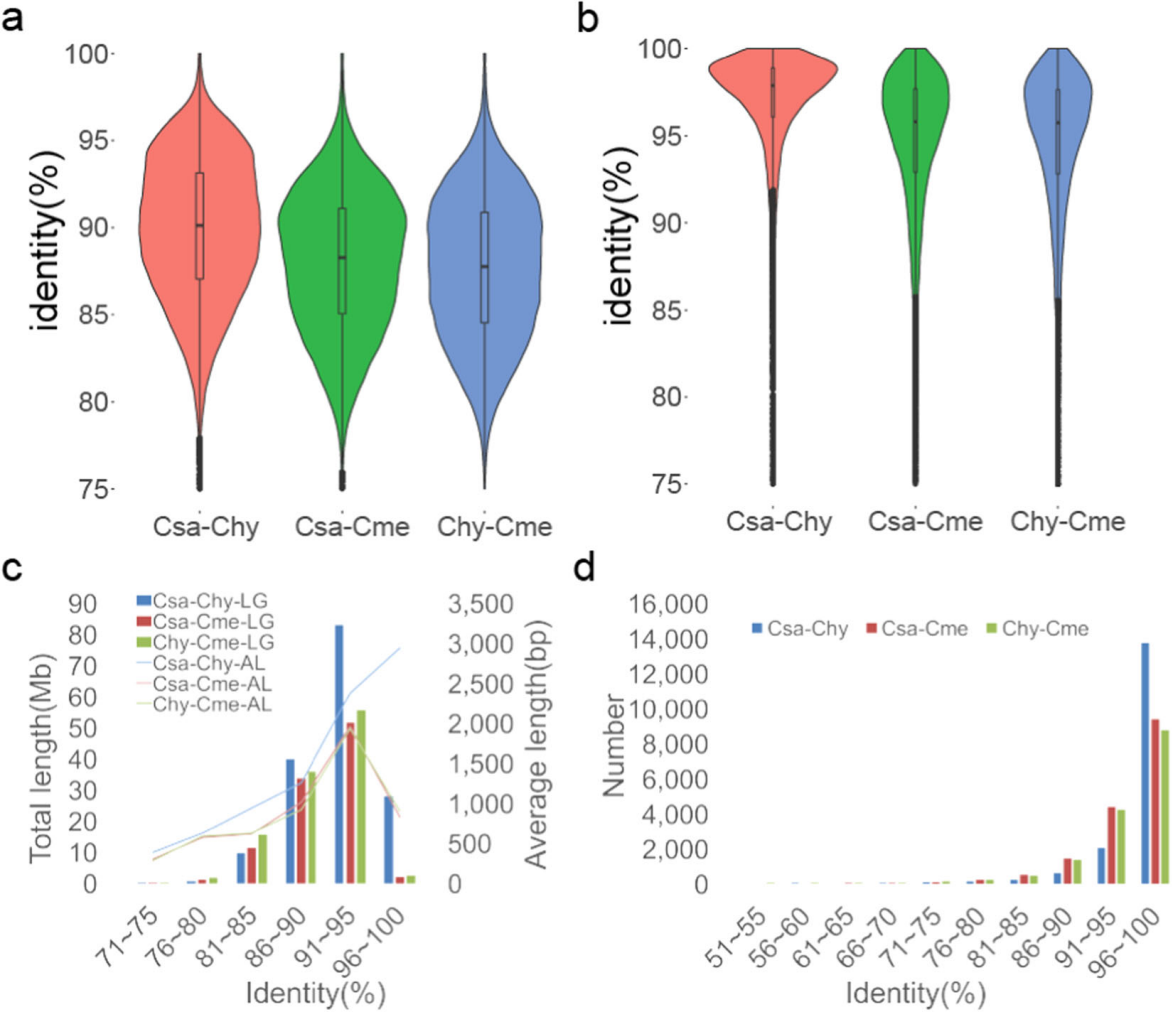
Similarities among cucumber, *Cucumis hystrix*, and melon at the nucleotide and protein levels. **a** Identity distribution of the aligned segments with one-to-one correspondence. **b** Identity distribution of pairwise best-hit proteins. **c** Total and average length of each identity interval. **d** Total number of pairwise proteins in each identity interval. LG, length; AL, average length; Csa, *Cucumis sativus*; Chy, *Cucumis hystrix*; Cme, *Cucumis melo*

We further examined the identity distribution of sequences showing one-to-one correspondence (Fig. 2a) and calculated the total and average length of the aligned sequences in each identity interval (Fig. 2c). *C. hystrix* shared a higher similarity median with cucumber than with melon. The median between *C. hystrix* and melon was low and that between cucumber and melon was comparable (Fig. 2a). *C. hystrix* and cucumber shared the most genomic sequences with high similarity (above 85%). *C. hystrix* shared a longer average length of aligned sequences in each identity interval with cucumber than with melon (Fig. 2c). In addition, *C. hystrix* shared a higher average identity of protein reciprocal best hits (RBHs) with cucumber (96.56%) than with melon (94.34%), and the average identity of RBHs between cucumber and melon was moderate (94.41%). The similarity distribution of RBHs demonstrated that *C. hystrix* shared a significantly higher median with cucumber than with melon (Fig. 2b), and most proteins showed over 95% similarity (Fig. 2d). The higher similarity of *C. hystrix* with cucumber at the DNA and protein level explained their close relationship and the cucumber-like phenotype of *C. hystrix*, providing a molecular basis for the successful hybridization between these two species.

### Genome collinearity of cucumber, *C. hystrix*, and melon

**Fig. 3:**
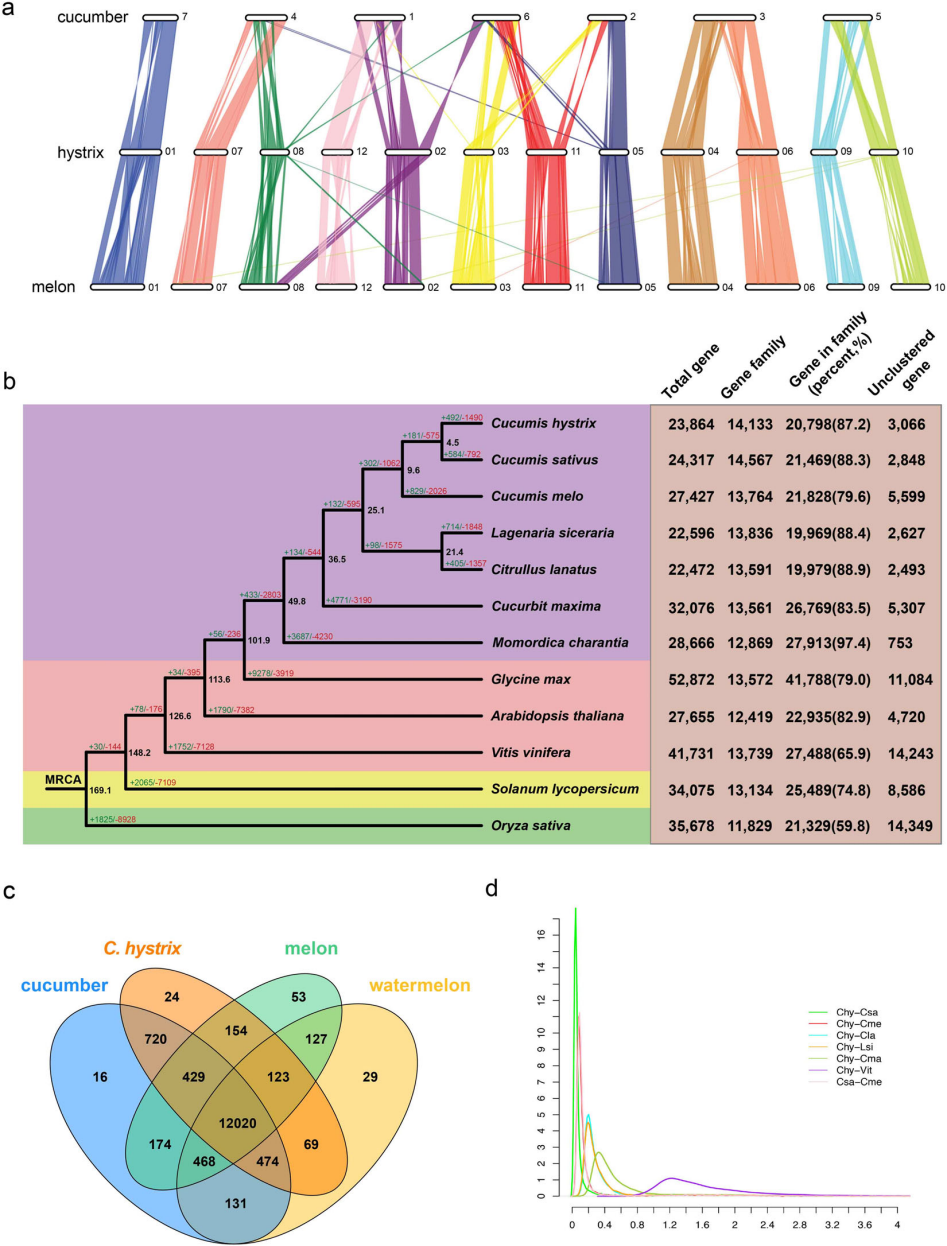
Genome evolution of *Cucumis hystrix*. **a** Genome collinearity analysis of cucumber, *Cucumis hystrix*, and melon. Chromosome number is showed at the right end of each chromosome diagram. **b** Phylogenetic relationships of the 12 selected species and gene family evolution. The numbers of total genes, gene families, clustered genes, and unclustered genes are summarized in the right table. Black numbers at each node represent the estimated time of each divergent event. Green and red numbers along each branch indicate the number of expanded and contracted gene families, respectively. MRCA, most recent common ancestor. **c** Venn diagram of shared gene families among cucumber, *C. hystrix*, melon, and watermelon. **d** Ks distribution of synthetic orthologs of the selected species. Csa, *C. sativus*, *C. hystrix*, Cme, *C. melo*; Cla, *Citrullus lanatus*; Lsi, *Lagenaria siceraria*; Cma, *Cucurbita maxima*; Vit, *Vitis vinifera*

We detected 16,916 RBHs between cucumber and *C. hystrix*, 16,131 RHBs between *C. hystrix* and melon, and 15,200 RHBs between cucumber and melon. We then used these RBHs to assess the collinearity among the three *Cucumis* species using McScanX^30^. Respectively, 119, 240, and 182 blocks with at least 5 RBHs were detected between *C. hystrix* and cucumber, *C. hystrix* and melon, and cucumber and melon. The average number of gene blocks between cucumber and *C. hystrix* was 137, almost two-fold the number between *C. hystrix* and melon (79) and more than two-fold the number between cucumber and melon (64). The largest block with the highest number of genes was also detected between cucumber and *C. hystrix*, which contained 960 orthologous gene pairs and covered 10.8 Mb genomic sequences of *C. hystrix* on chromosome 6 and 9.4 Mb genomic sequences of cucumber on chromosome 3. The statistics of RBHs and the detected blocks are summarized in Table S6. Detailed information of each block is presented in Tables S7–S9. Based on the position of the blocks detected, the overall collinearity across the whole genomes of the three *Cucumis* species is demonstrated in Fig. 3a. The primary syntenic relationship of the chromosomes was highly consistent with the previous reports^27^, detected by the comparison of linkage maps. *C. hystrix* showed the same karyotype as melon, but it shared fewer blocks and more average genes per block with cucumber microscopically, although their collinear blocks showed a complex, mosaic correspondence. These results indicate the occurrence of recent large-scale chromosomal rearrangements, which likely played a key role in cucumber speciation. Moreover, phylogenetic analyses based on the overall collinearity or robust karyotypes of species yield unreliable results.

### Phylogenetic tree and specific or expanded/contracted gene families in *Cucumis* species

We clustered genes of the three *Cucumis* species, four non-*Cucumis* Cucurbitaceae species (bottle gourd, watermelon, squash, and bitter gourd), and five other species, including rosids (soybean, *Arabidopsis*, and grape), asterids (tomato), and monocots (rice), into 17,901 gene families using OrthoFinder^31^. The numbers of total genes, gene families, clustered genes, and unclustered genes are listed in the right orange table of Fig. 3b. We focused on the gene families of *Cucumis* species using watermelon as the outgroup. General statistics are presented as a Venn diagram (Fig. 3c). A total of 15,011 gene families with at least two genes were clustered, and the four selected Cucurbitaceae species shared 12,020 gene families. Cucumber, *C. hystrix*, and melon shared 12,449 gene families, which could be recognized as the core gene set of *Cucumis* species. A total of 429 clusters were specifically shared by *Cucumis* species. *C. hystrix* shared the most gene families with cucumber, reflecting their close relationship. Moreover, 24 clusters containing 64 genes were unique to *C. hystrix*.

We collated 304 single-copy genes of the 12 species into supergenes to construct a phylogenetic tree (Fig. 3b). *C. hystrix* was the closest relative of cucumber, and their common ancestor was placed in the same clade as melon, which is consistent with previous reports^19^. We then calculated the synonymous substitution rate of each collinear paralogous gene between and within several selected species. The density distribution indicated that *C. hystrix* shared the smallest peak with cucumber (Fig. 3d). We further estimated that *C. hystrix* and cucumber diverged from their common ancestor about 4.5 million years ago (mya), indicating that they had a relatively short divergence time.

Gene family expansion and contraction play significant roles in phenotypic adaption during speciation. Duplicated genes may enhance the metabolic pathways in which they participate and may also acquire novel functions—called neofunctionalization^32–34^. We conducted gene family expansion and contraction analysis of the shared gene families among the 12 selected species (Fig. 3b). There were 584/792, 492/1490, and 829/2026 expanded/contracted gene families in cucumber, *C. hystrix*, and melon, respectively. The top 20 Gene Ontology (GO) enrichment terms of the expanded gene families for each *Cucumis* species are shown in Fig. S4. The most enriched and abundant function in *C. hystrix* was “defense response” (GO:0006952), which might protect this species from various abiotic or biotic stresses in the wild. “Organelle organization” (GO:0006996) was the most enriched function and “developmental process” (GO:0032502) was the most abundant function in cucumber. “DNA integration” was the most enriched function (GO:0015074) and “cellular metabolic process” (GO:0044237) was the most abundant function in melon. No overlap in function was noted among the expanded gene families of *Cucumis* species, indicating that their expansion may have driven *Cucumis* speciation.

### Positively selected genes (PSGs) in *C. hystrix*

We identified 55, 121, and 92 PSGs in cucumber, *C. hystrix*, and melon, respectively (false discovery rate <0.05), using PosiGene^35^. Here, we focus on the PSGs in *C. hystrix*. We found that 93 (76.9%) PSGs were single-copy, which likely played important roles in *C. hystrix* speciation. We further conducted GO analysis of these PSGs and observed 18 enriched PSGs (Table 2). Two of these enriched processes were “response to biotic stimulus” (GO:0009607) and “defense response to other organisms” (GO:0098542), involving 12 genes, which likely enhanced the disease resistance of *C. hystrix*. For instance, the homolog of *ChyUNG234630.1* in *Arabidopsis thaliana* (AT5G06720 and AtPRX53), which plays diverse roles in wound response, flower development, and syncytium formation, was found to be involved in response to nematode infection in soybean^36^ and *A. thaliana*^37^. Moreover, the homolog of *Chy3G060900.1* in *A. thaliana* (AT2G45180 and DRN1), a nonspecific lipid transfer protein, was found to be essential for resistance against various phytopathogens and tolerance to salt stress^38^. The general information of these 12 genes is summarized in Table S10.

**Table 2:** Enriched Gene Ontology (GO) terms for positively selected genes in *Cucumis hystrix*

GO ID	Description	*P* value
GO:0015691	Cadmium ion transport	0.000971
GO:0051351	Positive regulation of ligase activity	0.001243
GO:0051443	Positive regulation of ubiquitin-protein transferase activity	0.001243
GO:0032973	Amino acid export	0.001885
GO:0031398	Positive regulation of protein ubiquitination	0.002253
GO:1903322	Positive regulation of protein modification by small protein conjugation or removal	0.002653
GO:0009605	Response to external stimulus	0.004474
GO:1901658	Glycosyl compound catabolic process	0.004554
GO:0098542	Defense response to other organisms	0.004660
GO:0006218	Uridine catabolic process	0.005996
GO:0018160	Peptidyl-pyrromethane cofactor linkage	0.005996
GO:0090228	Positive regulation of red or far-red light signaling pathway	0.005996
GO:0008654	Phospholipid biosynthetic process	0.006707
GO:0046474	Glycerophospholipid biosynthetic process	0.006939
GO:0051340	Regulation of ligase activity	0.007586
GO:0051438	Regulation of ubiquitin-protein transferase activity	0.007586
GO:0009607	Response to biotic stimulus	0.008847
GO:0072527	Pyrimidine-containing compound metabolic process	0.008851

### Identification of resistance (R) gene analogs (RGAs) and evolutionary analysis of nucleotide-binding site (NBS)-encoding genes in *Cucumis*

**Fig. 4:**
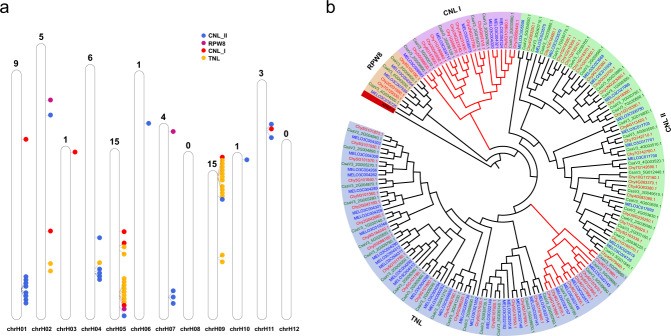
Nucleotide-binding site (NBS)-encoding gene families of cucumber, *Cucumis hystrix*, and melon. **a** NBS gene distribution on each *C. hystrix* chromosome. The total number of NBS genes on each chromosome is labeled above. Dots with different colors represent different types of NBS genes. **b** Phylogenetic tree of NBS genes in cucumber, *C. hystrix*, and melon. Expanded clade/subclade in *C. hystrix* is labeled in red. Gene IDs of cucumber, *C. hystrix*, and melon are labeled in green, red, and blue, respectively. *APAF-1* was used as the outgroup

The R genes play critical roles in the arms race of plant–pathogen interaction in the immune system of plants^39^. We used RGAugury^40^ to identify the potential RGAs in the three *Cucumis* species. The total predicted RGA numbers for each species are listed in Table 3. Here, we focused on the R genes containing the NBS domain, which are the most frequently cloned and described genes in plants^41,42^. We detected 74, 104, and 84 RGAs in cucumber, *C. hystrix*, and melon, respectively. Genes with <80% coverage of the NBS domain were excluded from the subsequent analysis, finally yielding 54, 65, and 51 genes. We anchored each NBS-encoding gene (excluding the genes on scaffolds) of *C. hystrix* to its pseudochromosomes (Fig. 4a). The results indicated that 39 (60%) NBS-encoding genes were located on chromosomes 1, 5, and 9, with most exhibiting a clustered pattern, which is consistent with previous reports^43–45^. The remaining chromosomes were sporadically distributed on other chromosomes. There were no full-length NBS-encoding genes predicted on chromosomes 8 and 12.

**Table 3:** Number of resistance genes in the three *Cucumis* species

Species	NBS encoding								RLP	RLK	TM-CC
	NBS	CNL	TNL	CN	TN	NL	TX	Other			
*C. sativus*	5	16	15	3	3	21	5	6	49	436	122
*C. hystrix*	20	23	16	8	2	18	7	10	55	347	130
*C. melo*	11	13	17	5	5	19	9	5	40	359	103

*CC*, coiled-coil; *LRR*, leucine-rich repeat; *LysM*, lysin motif; *NB-ARC*, nucleotide binding-activity regulated cytoskeleton; *NBS*, nucleotide-binding site; *RGA*, resistance gene analog; *RLK*, receptor-like kinase; *RLP*, receptor-like protein; *STTK*, serine/threonine and tyrosine kinase; *TIR*, Toll/interleukin-1 receptor; *TM*, transmembrane; *CNL*, CC-NBS-LRR; *TNL*, TIR-NBS-LRR; *CN*, CC-NBS; *TN*, TIR-NBS; *NL*, NBS-LRR; TX, TIR-unknown domain; Other, CC or TIR; RLP, TM-LRR, TM-LysM; RLK, TM-LRR-STTK, and TM-LysM-STTK

To study the evolution of the predicted genes containing the full-length NBS domain in the *Cucumis* species, we constructed a phylogenetic tree using the sequences of the conserved NB-ARC (PF00931) domain (Fig. 4b). The sequences formed four main clusters, namely RPW8, CNL I, CNL II, and TNL. RPW8 was the smallest cluster, with three genes in each *Cucumis* species. The CNL I cluster was significantly expanded in *C. hystrix* (11), containing almost two-fold more genes than in cucumber (6) and melon (6). The number of genes in the CNL II cluster was comparable between cucumber (22) and *C. hystrix* (25), but the number in melon (13) was half the number in the other two species. The number of genes in the TNL cluster was comparable among the three *Cucumis* species, being 23 in cucumber, 26 in *C. hystrix*, and 29 in melon. Moreover, a subclade of TNL was expanded in *C. hystrix* (Fig. 4b). In addition, the TNL cluster was located between two clusters on chromosomes 5 and 9, and the CNL II cluster between two clusters on chromosomes 1 and 4. The expanded NBS-encoding genes in *C. hystrix* might explain its high disease resistance to some extent.

### Development and identification of cucumber—*C. hystrix* alien additional lines (CH-AALs)

AALs are powerful tools for genome structure research and functional genomics and may serve as a bridge to introgress useful genes into recurrent parents in crop breeding. We developed four CH-AALs with different *C. hystrix* chromosomes by recurrently backcrossing the artificial allotetraploid to cucumber. The detailed process is illustrated in Fig. S5. These lines were morphologically distinct, and the typical phenotype of each CH-AAL is shown in Fig. S6.

**Fig. 5:**
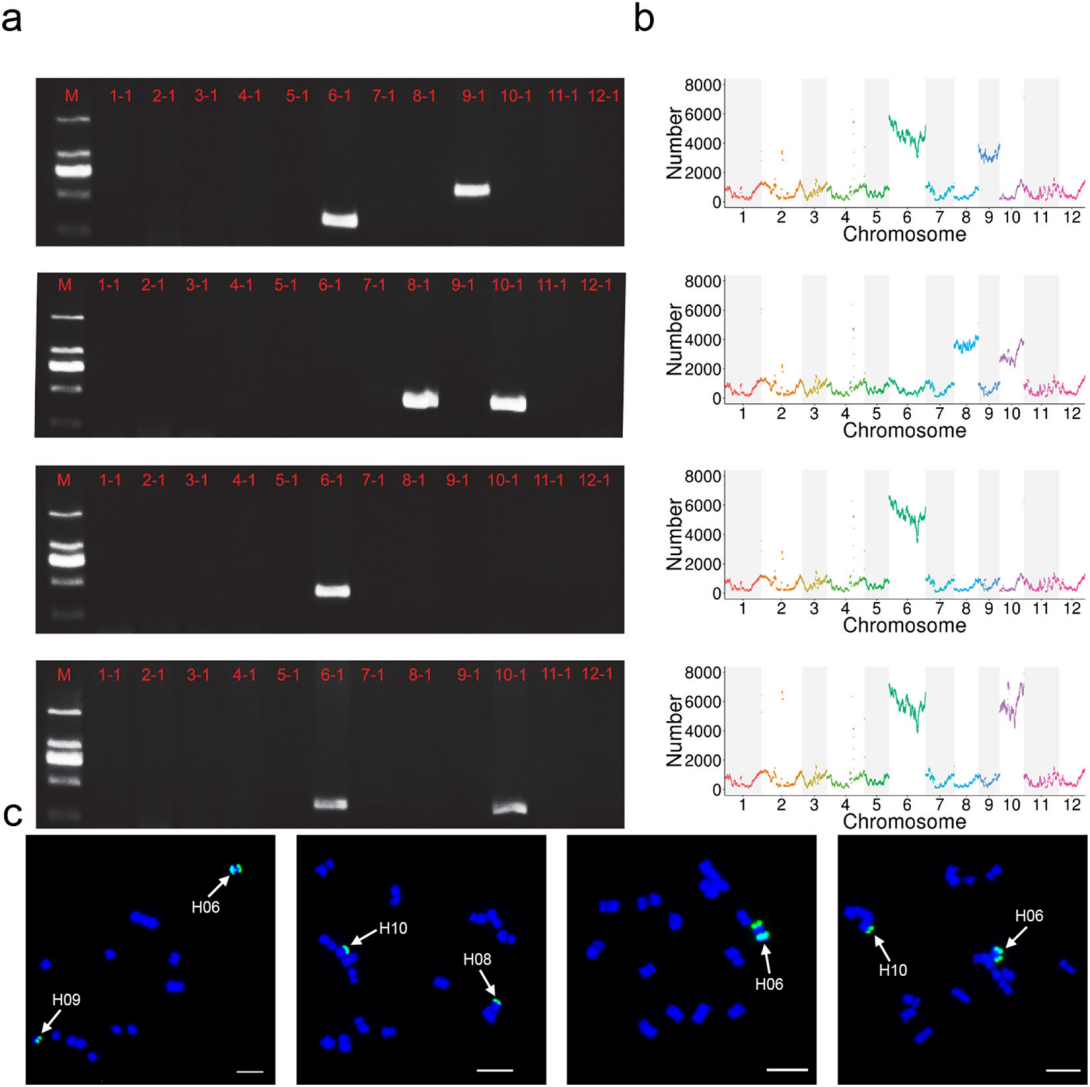
Verification of the exact identity of each alien chromosome in cucumber—*C. hystrix* alien additional lines (CH-AALs). **a** Specific polymerase chain reaction band(s) of selected primer pair(s) for each CH-AAL (CH-AAL01–CHAAL04, from top to bottom). **b** Number of highly similar reads aligned to the *C. hystrix* genome in sliding windows (1 Mb in size) for each CH-AAL (CH-AAL01–CH-AAL04, from top to bottom). **c** Fluorescence in situ hybridization signal of introgressed *C. hystrix* chromosome(s) in each CH-AAL (CH-AAL01–CH-AAL04, from left to right)

To verify the exact identity of each alien chromosome in each CH-AAL, we first developed chromosome-specific markers for *C. hystrix* and performed polymerase chain reaction (PCR) for each line. A total of 45,417 chromosome-specific sequences of *C. hystrix* were identified through inter- and intraspecific whole-genome alignment, ranging from 28 to 59,678 bp. Of these, 9218 sequences were over 400 bp and evenly distributed on each chromosome (Fig. S7a). Chromosome-specific sequences of cucumber were also identified and found to be evenly distributed on each chromosome (Fig. S7b). We selected 36 *C. hystrix* chromosome-specific sequences as markers (three on each chromosome) to design primers (Table S11). We conducted PCR for *C. hystrix* and cucumber, and all selected markers produced a chromosome-specific band in *C. hystrix* (Fig. S8). We selected one marker from each chromosome to conduct PCR for all CH-AALs (Fig. 5a). CH-AAL01 specifically produced bands for chrH06 and chrH09 (Fig. 5a, first from top). CH-AAL02 specifically produced bands for chrH08 and chrH10 (Fig. 5a, second from top). CH-AAL03 produced a single band from chrH06 (Fig. 5a, third from the top). CH-AAL04 produced bands for chrH06 and chrH10 (Fig. 5a, fourth from top). The chromosome-specific bands produced by each CH-AAL reflected introgression of *C. hystrix* segments into cucumber.

We further confirmed the identity of the alien chromosomes using next-generation sequencing (NGS) and fluorescence in situ hybridization (FISH). NGS reads (150 bp read length) of each CH-AAL were aligned to the *C. hystrix* genome, and the number of highly similar reads (>99% identity with an alignment length of at least 145 bp) in each sliding window was determined (Fig. 5b). Chromosomes chrH06 and chrH09 were covered by a large number of highly similar reads showing a continuous pattern in CH-AAL01 (Fig. 5b, first from top). FISH signals of chrH06 and chrH09 were also detected in this line (Fig. 5c, first from left; Fig. S9a, b). The NGS and FISH results were consistent with the PCR results. Therefore, we confirmed that CH-AAL01 received chrH06 and chrH09 from *C. hystrix*. The identity of the introgressed *C. hystrix* chromosomes in the remaining three CH-AALs was also verified using the same method (Fig. 5b, c and Fig. S9), and the detailed process is described in Materials and methods. All NGS and FISH results were consistent with the corresponding PCR results. Collectively, we successfully verified the exact identity of each *C. hystrix* chromosome in all CH-AALs using different methods. The developed chromosome-specific markers may be used to efficiently screen for additional interspecific materials between *C. hystrix* and cucumber, serving as a bridge to enrich the cucumber gene pool.

## Discussion

Phylogenetic relationships are key factors in determining the success of interspecific hybridization and the efficiency of genetic material exchange (introgression)^46,47^. *C. hystrix* has a 2n = 2x = 24 karyotype—the same as melon—and they generally show a good genome collinearity. Meanwhile, cucumber has a distinct 2n = 2x = 14 karyotype. However, we found that *C. hystrix* shares better synteny with cucumber. The overall chromosome correspondence among the three *Cucumis* species tested in this study corroborated the previous reports^27^. Furthermore, we confirmed that *C. hystrix* is phylogenetically closer to cucumber than to melon at the molecular level based on the results of comprehensive genome-scale analysis, which explains the cucumber-like phenotype of *C. hystrix*. These findings further indicate that phylogenetic relationships based on karyotypes or overall collinearity can be misleading, and it is better to construct a robust phylogenetic tree at the molecular level to clarify the relationships among species, which is of high value for evolutionary studies and interspecific breeding. In addition, large-scale chromosome rearrangements, such as Robertsonian translocation, can drive speciation^48^. The complex events that shaped the evolution of seven pairs of chromosomes in cucumber from the 12 ancestral ones likely occurred gradually. However, this gives rise to other questions—were there any other phylogenetically intermediate species between *C. hystrix* and cucumber, and if so, do they still exist? It would be interesting and important to explore the answers, which would benefit the evolutionary studies and introgression breeding of *Cucumis* species.

Crops originate from their wild ancestors through domestication, during which artificial selection acts as a powerful driver shaping the crop genomes as well as their morphological characteristics and growth habits beneficial to humans^49^. The genetic base of cucumber, an economically important vegetable crop, has become extraordinarily narrow due to long-term domestication and recurrent use of limited variation during breeding^6^. As opposed to melon, which has been independently domesticated multiple times and has numerous cross-fertile wild ancestors with a wide distribution from Asia to Africa^50^, cucumber has a single cross-fertile wild ancestor originating from India, named *C. sativus* var. *hardwickii*, and the domestication of cucumber is limited to India^19^. Thus, cucumber breeding based only on intraspecific variation has encountered a bottleneck. In this light, successful interspecific hybridization of cucumber with its close wild relative *C. hystrix* provides an excellent opportunity to introgress novel genes, specifically those related to biotic or abiotic stress responses, in cucumber. In this study, we conducted comparative genomic analysis of cucumber, *C. hystrix*, and melon and demonstrated that gene families involved in defense response (e.g., NBS-LRR) have significantly expanded in *C. hystrix* compared to those in cucumber and melon. A considerable number of PSGs in *C. hystrix* responded to biotic stimuli compared to those in the other selected Cucurbitaceae species. Finally, we developed and verified four phenotypically distinct cucumber lines introgressed for different *C. hystrix* chromosomes, which may serve as a bridge for introgressing novel genes from *C. hystrix* to cucumber.

Crop breeding has entered a new era in which genomic information has become increasingly pivotal^51,52^. In this study, we developed numerous chromosome-specific markers through the assembly of *C. hystrix* draft genome. We verified the specificity of these markers and found that they were evenly distributed on each *C. hystrix* chromosome, which could be of great significance for efficiently and unambiguously tracing the segments introgressed from *C. hystrix* to cucumber. Collectively, our findings provide valuable resources and data for evolutionary studies on *Cucumis* and lay a foundation for efficient cucumber breeding via interspecific hybridization.

## Materials and methods

### Plant material, DNA and RNA extraction, and sequencing

Seeds of *C. hystrix* were collected by Professor Jinfeng Chen from Xishuangbanna (Yunnan, China) and self-pollinated for several generations by germinating on Petri dishes at 25 °C. High-quality DNA was extracted from fresh young leaves using a modified cetyltrimethylammonium bromide method. A 10× Genomics Chromium library was constructed according to the manufacturer’s instructions within droplets containing Gel Beads-in-Emulsion (GEMs) mixed with DNA and polymerase for whole-genome amplification. DNA was sheared within each GEM, and each molecule was tagged with an identical barcode (linked reads). As a result, 35 Gb reads with a length of 150 bp were generated by sequencing the library on the Illumina HiSeq X Ten platform. One pair-end with an insert size of 500 bp and four mate-pair “jumping libraries” with insert sizes of 2 and 8 k were constructed following the standard Illumina protocol. The reads were sequenced on the Illumina Hiseq 2500 platform, and 27 Gb of pair-end (read length, 250 bp) and 49 Gb of mate-pair (read length, 125–150 bp) sequences were generated. For PacBio data sequencing, the genomic DNA was sheared into segments of 15–40 kb, and a single-molecule real-time library was constructed following the PacBio-recommended method. We obtained 10 Gb of PacBio sequences with an average length of 5.6 kb. The corresponding statistics are summarized in Table S1.

RNA from five *C. hystrix* tissues (root, stem, leaf, ovary, and male flower) was extracted using the QIAGEN RNeasy Plant Mini Kit, following the manufacturer’s instructions (QIAGEN, Valencia, CA, USA). Strand-specific RNA-sequencing (RNA-Seq) libraries were constructed using the protocol described by Zhong et al.^53^. The RNA-Seq libraries were sequenced on the Illumina HiSeq X system with a pair-end read length of 150 bp. We obtained 8.5, 9.3, 9.8, 10.6, and 9.6 Gb sequences from the five tissues, respectively.

The detailed process of CH-AAL development is presented in Fig. S5. The protocol for DNA sample preparation was the same as above. The libraries were constructed according to the manufacturer’s instructions. Resequencing of these libraries generated 8.5 (CH-AAL01), 9.2 (CH-AAL02), 10.6 (CH-AAL03), and 12.3 Gb (CH-AAL04) pair-end reads with a length of 150 bp on the Novaseq 6000 sequencing system.

### Genome assembly and quality assessment

The genome size, heterozygosity, and repeat content of the *C. hystrix* genome were estimated using GCE^54^. First, 10× genomic-linked reads were assembled using Supernova^28^. The read number used for assembly was calculated according to the recommended depth. We fed Pilon^55^ with the PE250 pair-end data, which were filtered by fastp^56^ according to the base quality, length, and overlapping information, to polish the scaffolds generated by Supernova. To fill the gaps in the polished scaffolds, we first assembled super-reads by running MaSuRCA^57^ on all raw PE reads. PacBio long reads were then self-corrected using Canu^58^. Super-reads and the corrected long reads were merged and fed to PBjelly^59^ for gap-filling. The Pilon polishing step was repeated on the gap-filled scaffolds. We ran SSPACE^60^ on the 2 and 8 k mate-pair libraries, which were filtered by NextClip^61^ to further merge the secondary polished scaffolds. We conducted e-PCR^62^ on the markers from the linkage group developed by Yang et al.^27^ to locate them on the scaffolds. Finally, based on the marker location information on the scaffolds and linkage groups, the scaffolds were anchored, ordered, and oriented along 12 pseudochromosomes using ALLMAPS^63^. The assembly workflow is summarized in Fig. S1.

We used three methods to evaluate the quality of our genome assembly. We first checked the consistency of the assembly with a linkage map using ALLMAPS. The mate-pair reads of the 2 and 8 k library were aligned to the assembly using the Burrows–Wheeler Aligner^64^, and a 2.5 Mb segment of chromosome 2 was selected as an example. We further examined the coding region completeness of the genome assembly and the other selected Cucurbitaceae species with BUSCO^29^.

### Genome annotation

We first detected the repeat sequences in the final assembly using RepeatModeler. De novo-detected repeats were then combined with the TIGR plant repeats database (http://plantrepeats.plantbiology.msu.edu) and repeated with RepeatMasker (http://repeatmasker.org).

A hybrid method of transcriptome mapping, ab initio, and homologous alignment was used for gene prediction of the repeat-masked assembly. Transcriptomic data from five tissues were mapped to the reference with HISAT2^65^ and assembled using stringtie^66^. The output transcripts were then fed to PASA (http://pasa.sourceforge.net) for further processing. Three tools, including GlimmerHMM^64^, Augustus^66^, and SNAP^67^, were used for ab initio prediction. Non-redundant plant proteins from Uniprot (http://www.uniprot.org) were downloaded and aligned to the assembly with Wise^67–70^. Finally, EVidenceModeler^71^ was used to integrate the evidence detected and generated gene structures based on their weights. The completeness of the final predicted gene set was evaluated using BUSCO^29^.

### Comparative genomics

The whole genomes *Cucumis* species were aligned using Mummer 4.0^72^ with default parameters. RBHs were identified using a script that depends on BLAST+^73^ and then fed to McScanX^30^ to detect syntenic blocks between each pair of species.

To calculate the synonymous substitution rate (Ks) of the homologous gene pairs in the selected species, we first conducted all-vs-all BLASTP (*E* value <1e−5). Collinear homologous gene pairs within or between species were identified using McScanX^30^. We then aligned their coding sequences (CDSs) using ParaAT^74^. Finally, the Ks value of each homologous gene pair was calculated using KaKs_Calculator^75^.

Orthofinder^31^ was used to identify gene families of *C. hystrix*, cucumber, and melon, as well as the selected 9 species, including four non-*Cucumis* Cucurbitaceae (bottle gourd, watermelon, squash, and bitter gourd), four other dicot species, including rosids (soybean, *Arabidopsis*, and grape) and asterids (tomato), and one monocot species (rice). Gene family expansion/contraction was detected with Café^76^ using a probabilistic graphical model. Next, 304 single-copy genes identified by OrthoFinder in the 12 aforementioned species were fed into RAxML^77^ to clarify their phylogenetic relationships. To estimate the divergence time of the species, we used the MCMCtree program of PAML^78^. GO enrichment analysis was performed on the OmicShare online platform (http://www.omicshare.com/tools).

PSGs of *C. hystrix* were identified by feeding the CDSs of nine Cucurbitaceae species, including cucumber, *C. hystrix*, melon, watermelon, bottle gourd, *Cucurbita maxima*, monk fruit, bitter gourd, and wax gourd, to PosiGene^35^. We used cucumber as the anchor species. RGAs were predicted by RGAugury^40^. NBS-encoding genes were then extracted for further analysis. Genes with over 80% coverage of the NB-ARC (PF00931) domain were aligned using MUSCLE^79^. To illustrate the evolutionary history of the full-length NBS-encoding genes of the three *Cucumis* species, we constructed a phylogenetic tree using IQ-TREE^80^. The resulting Newick tree was fed to iTOL^81^ for visualization and further editing.

### Genome data collection

The genome data of cucumber, melon, watermelon, bottle gourd, *C. maxima*, and wax gourd were downloaded from the Cucurbit Genomics Database (http://cucurbitgenomics.org). The data of *Luffa cylindrical*^82^ and *Momordica charantia*^83^ were downloaded according to the corresponding reference. The cucumber genome version 3 and the melon genome version 3.5.1 were used in comparative genomics. Other genomic data were downloaded from the NCBI database.

### Identification of CH-AALs

For the amplification of *C. hystrix*-specific molecular markers, we first extracted the unmatched sequences of *C. hystrix* from its alignment with the cucumber genome. These species-specific sequences were then realigned to the *C. hystrix* genome using BLASTN with default parameters. Sequences showing no hits with other chromosomes were recognized as chromosome-specific markers. We selected three markers evenly distributed on each chromosome to verify their specificity using PCR (Fig. S8). Twelve markers, one from each chromosome, were used for PCR of the CH-AALs.

#### Analysis of the NGS data of CH-AALs

We first selected ~2× reads from the generated NGS data of each CH-AAL and aligned these to the *C. hystrix* draft genome using BLASTN (*E* value <1e−5). The best hit of each read was extracted from the BLASTN results. Reads with an alignment length >145 bp and sequence similarity above 99% were considered to be from *C. hystrix*. Finally, the number of reads from *C. hystrix* in each 1 Mb window with a step size of 10 kb was counted and visualized with an in-house R script.

#### FISH

We used the whole-genome DNA of *C. hystrix* as probes to conduct FISH in each CH-AAL and found one or two signals in all lines (Fig. 5c). To further verify the identity of the alien chromosomes, we designed different schemes. There were two alien chromosome signals in CH-AAL01 (Fig. 5c, first left). We used the oligo-probe pool of chromosome 5 (oligo C5) from cucumber^84^ to conduct FISH and found that one of the alien chromosomes showed a signal (Fig. S9a). Chromosome 5 of cucumber corresponded to chromosomes 9 and 10 of *C. hystrix* (Fig. 3a). According to our previous FISH results, only chromosomes 8, 10, and 12 showed 45S signals in *C. hystrix*^85^. Because this chromosome showed no 45S signal (Fig. S9a), we concluded that it was chromosome 9 from *C. hystrix*. Collinearity analysis in this study (Fig. 3a) demonstrated that a 6–6.5 Mb region of chromosome 3 of cucumber corresponded to a segment of chromosome 6 of *C. hystrix* (to clearly show collinearity, we reversed chromosome 3 of cucumber in Fig. 3a). We designed oligo probes for this region (oligo C3-a) from cucumber to conduct FISH in CH-AAL01. Another alien chromosome showed a hybridization signal (Fig. S9b), which was determined to be chromosome 6 of *C. hystrix*. CH-AAL02 showed two alien chromosome signals (Fig. 5c, second from left). We used the oligo-probe pool of chromosome 4 from cucumber (oligo C4)^86^ to conduct FISH and found that one of them showed a signal (Fig. S9c). Chromosome 4 of cucumber corresponded to chromosomes 5, 7, and 8 of *C. hystrix* (Fig. 3a). Because this alien chromosome showed a 45S signal (Fig. S9c), we concluded that it was chromosome 8 from *C. hystrix*. The oligo C5 of CH-AAL02 showed a signal in another alien chromosome (Fig. 9d) but no 45S signal (Fig. S9c). Therefore, it was determined to be chromosome 10 from *C. hystrix*. The oligo C3-a of CH-AAL03 showed one alien chromosome signal (Fig. 5c, second from right), which was determined to be chromosome 6 from *C. hystrix* (Fig. S9e). CH-AAL04 showed two alien chromosome signals (Fig. 5c, right), and one of them was a C3-a signal (Fig. S9f). The oligo C5 of CH-AAL04 showed a signal in another alien chromosome and a 45S signal (Fig. S9g), which were determined to be chromosomes 6 and 10 from *C. hystrix*, respectively. The protocols for probe synthesis and FISH have been described by Zhao et al.^84^ and Bi et al.^86^.

## Supplementary information

Revised Supplementary Figures

Revised Supplementary tables

## Data Availability

Raw sequencing reads used are deposited in the Sequence Read Archive database under the accession number PRJNA649392. The final genome assembly and annotation information can be downloaded at 10.6084/m9.figshare.13377671.
