# Supplementary material for: Chromosome-scale genome assembly of Cucumis hystrix—a wild species interspecifically cross-compatible with cultivated cucumber
Source: Hortic Res. 2021 Mar 1;8:40. doi: 10.1038/s41438-021-00475-5 (PMC7917098; doi:10.1038/s41438-021-00475-5)
Supplement: Supplementary file 1 — Revised Supplementary Figures [file 41438_2021_475_MOESM1_ESM.docx]

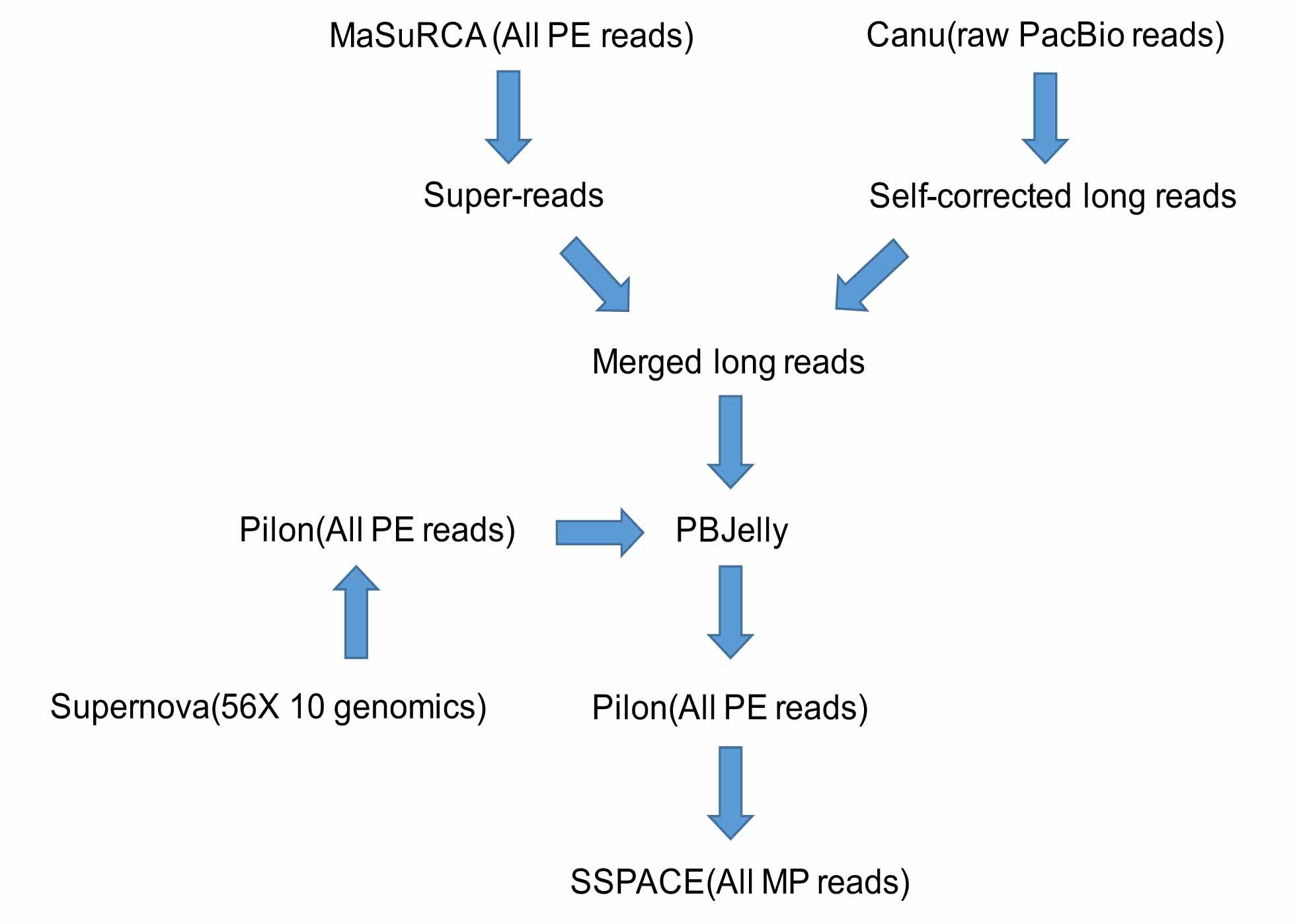


**Figure S1.** Workflow of the *de novo* assembly of *Cucumis hystrix*.


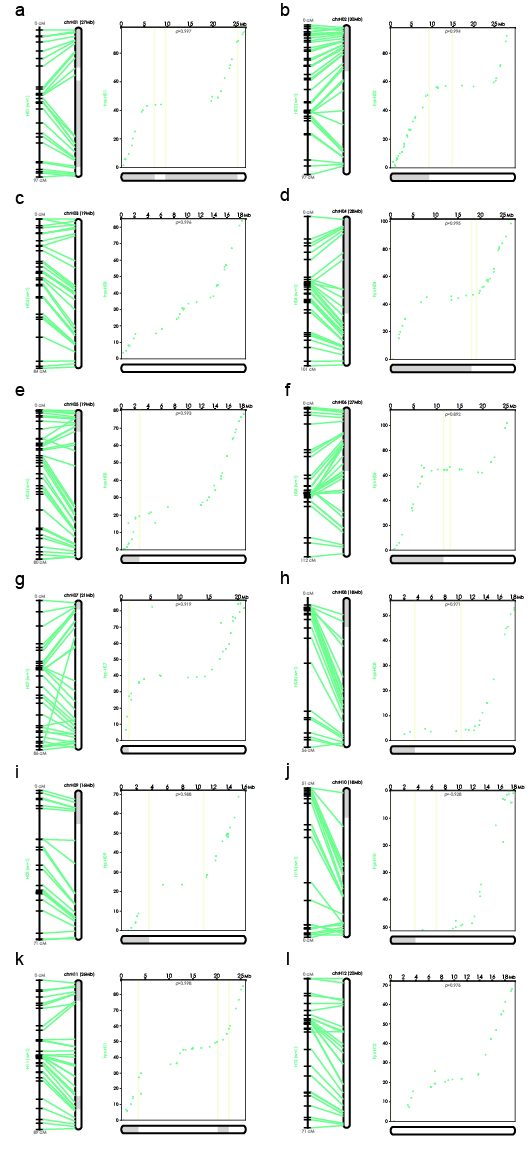


**Figure S2.** Consistency checking of each pseudochromosome to the corresponding linkage group.


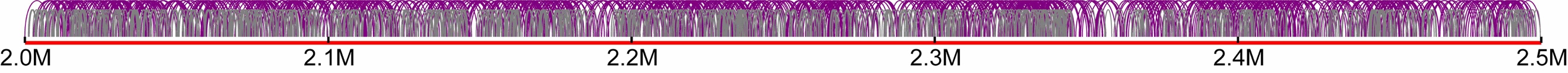


**Figure S3.** An example of checking the mate-pair reads of the genomic region of chrH02 (2.0 to 2.5 Mb).

Gray and purple arcs represent the libraries with an insert size of 2 and 8 kb, respectively.


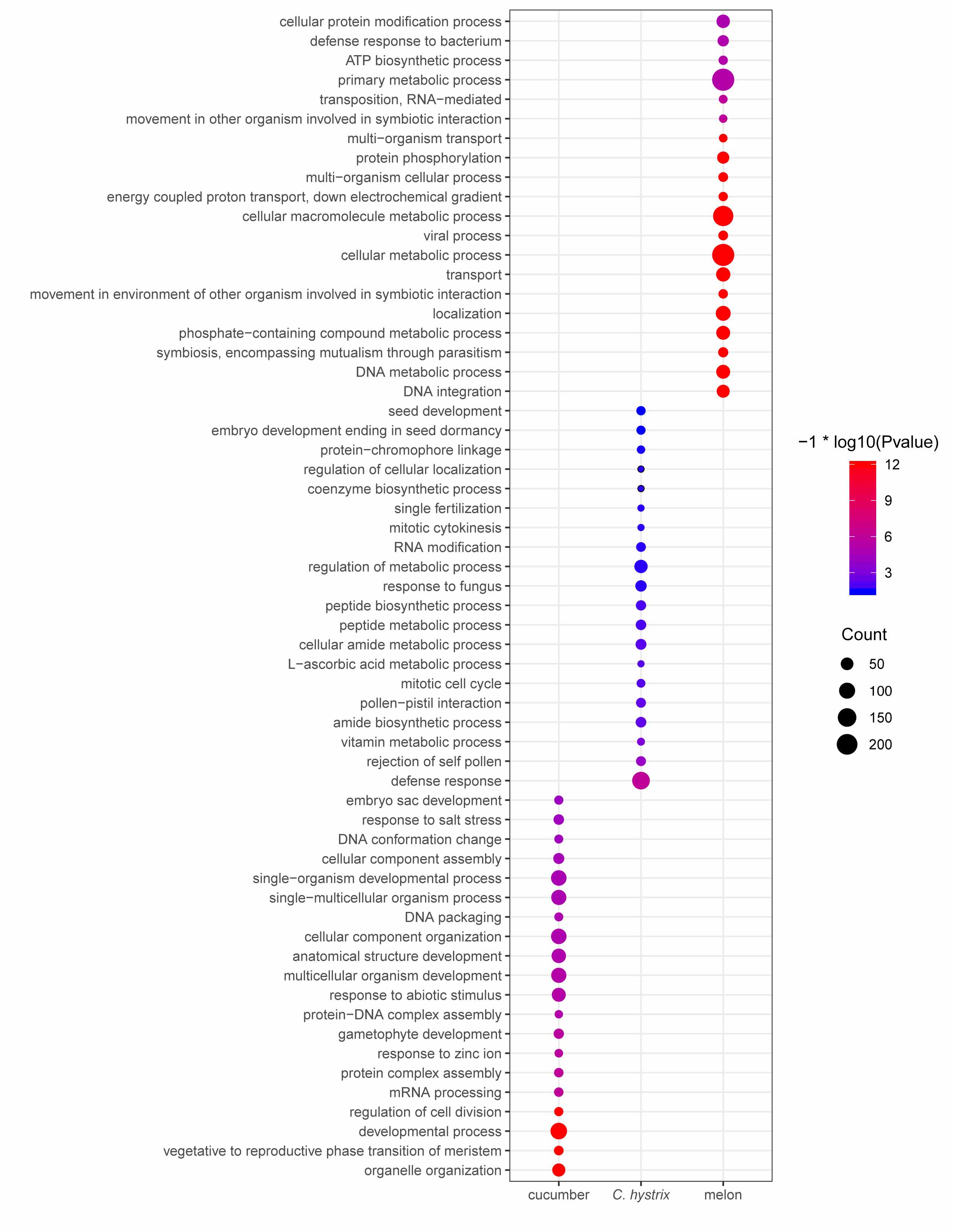


**Figure S4.** Gene Ontology enrichment of the expanded gene families of cucumber, *Cucumis hystrix*, and melon.

Circle colors from blue to red indicate significance, and the circle size corresponds to the gene number.


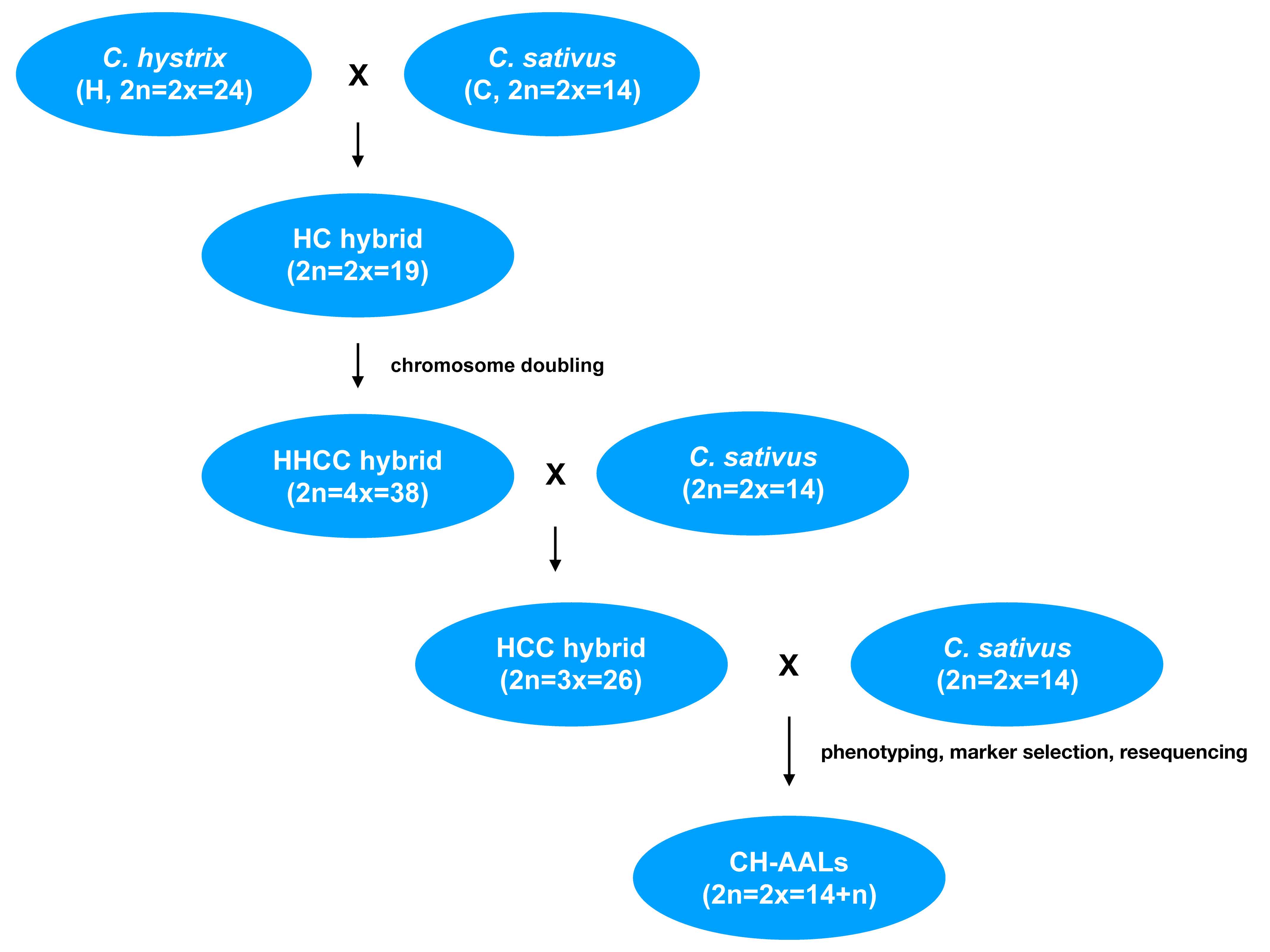


**Figure S5.** A flowchart of development of the cucumber–*Cucumis hystrix* alien addition lines.

**
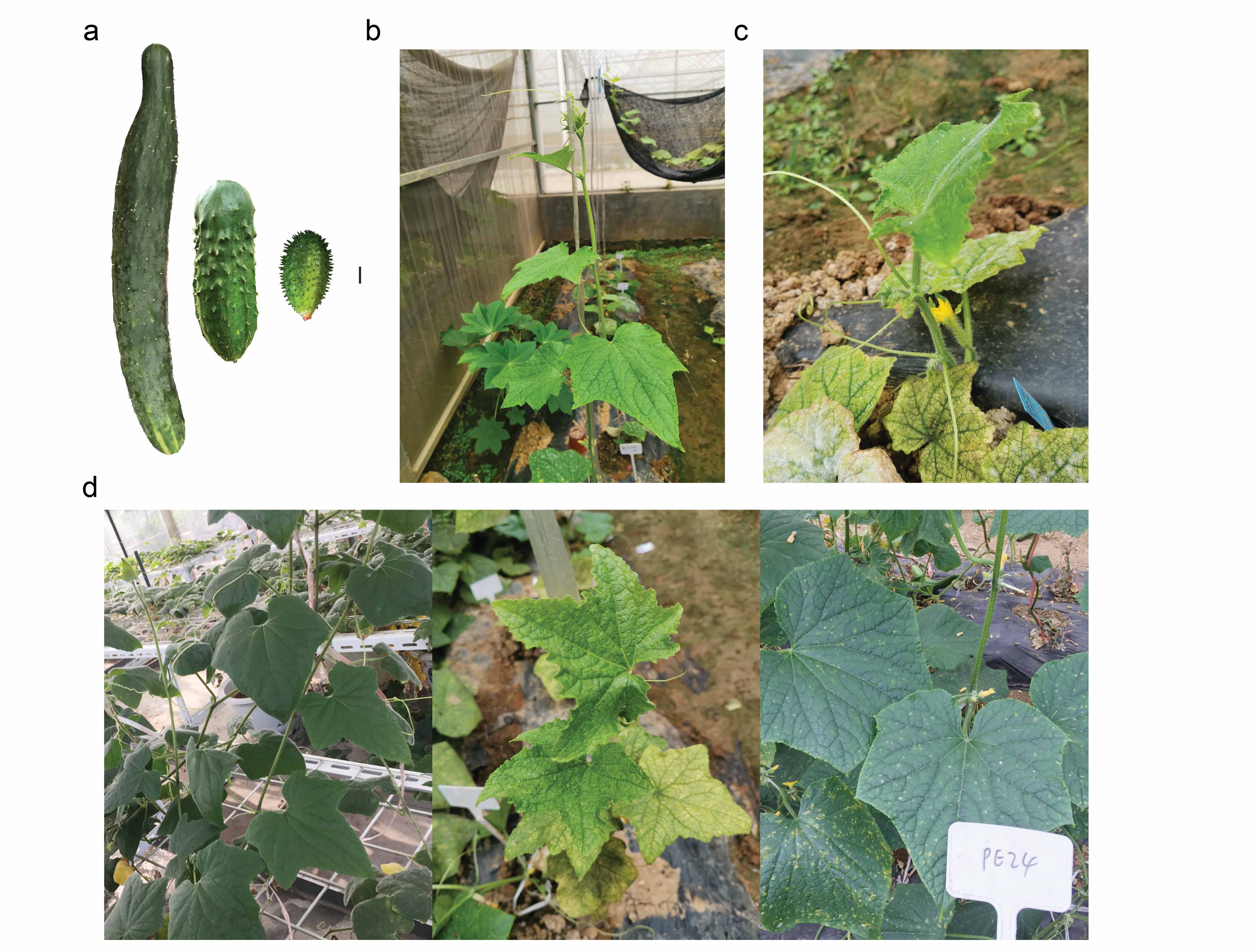
Figure S6.** Phenotype of each cucumber–*Cucumis hystrix* alien addition line (CH-AAL). (a) Fruits of cucumber, CH-AAL01, and *C. hystrix* (from left to right). (b) Stem tip of CH-AAL02. (c) Delayed development of CH-AAL03. (d) Leaves of *C. hystrix*, CH-AAL04, and cucumber (from left to right).


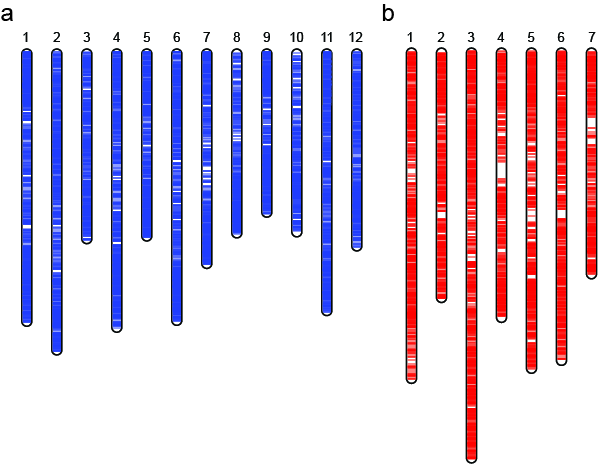


**Figure S7.** Distribution of chromosome-specific sequences of *Cucumis hystrix* and cucumber.


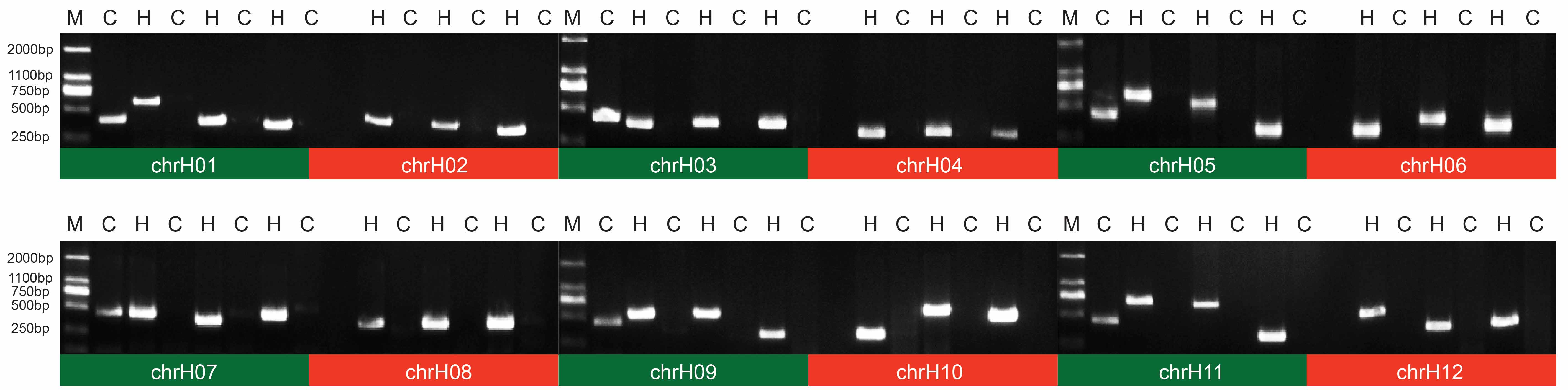


**Figure S8.** Chromosome-specific marker verification of *Cucumis hystrix.*

The first band obtained using a randomly selected primer confirmed that the cucumber DNA was not degraded.

.


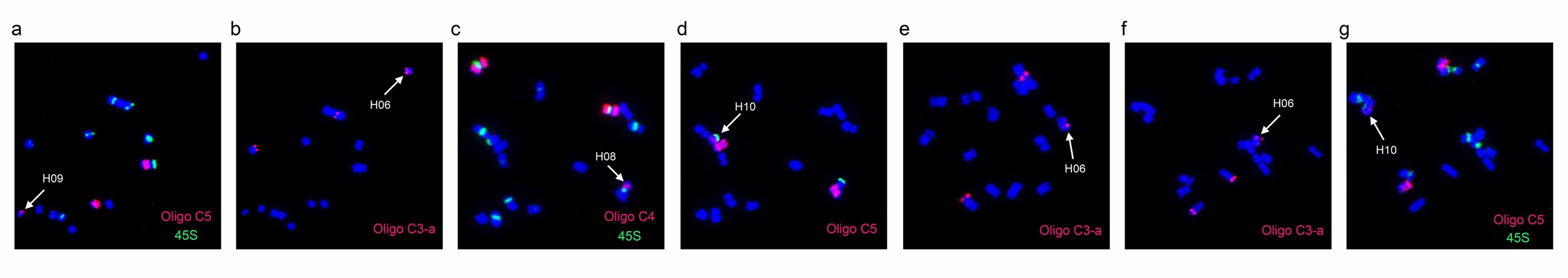


**Figure S9.** Verification of the identity of the additional chromosomes in the four additional lines using fluorescence *in situ* hybridization

The chromosomes introgressed from *Cucumis hystrix* are indicated by while arrows.
