# Supplementary material for: Chromosome-scale genome assembly of Cucumis hystrix—a wild species interspecifically cross-compatible with cultivated cucumber
Source: Hortic Res. 2021 Mar 1;8:40. doi: 10.1038/s41438-021-00475-5 (PMC7917098; doi:10.1038/s41438-021-00475-5)
Supplement: Supplementary file 2 — Revised Supplementary tables [file 41438_2021_475_MOESM2_ESM.docx]

**Table S1. Summary of data types and size used in assembly of the *Cucumis hystrix* genome**

| Library type | Insert size (bp) | Read length (bp) | Data size (Gb) | Depth |
| --- | --- | --- | --- | --- |
| Pair-end | 180 | 90 | 17 | 40 |
|  | 450 | 250 | 27 | 66 |
|  | 500 | 90 | 11 | 26 |
| Mate-pair | 2,000 | 150/125 | 24 | 58 |
|  | 8,000 | 150/125 | 25 | 61 |
| PacBio | - | 5,600* | 10 | 24 |
| 10X genomics | - | 150 | 35 | 87 |
| Total | - | - | 149 | 251 |

*average read length of PacBio data

**Table S2. Anchoring statistics of assembled scaffolds**

|  | Anchored | Oriented | Unplaced |
| --- | --- | --- | --- |
| Markers (unique) | 356 | 348 | 1 |
| Markers per Mb | 1.3 | 1.3 | 0.0 |
| N50 Scaffolds | 9 | 9 | 0 |
| Scaffolds | 35 | 29 | 2,256 |
| Scaffolds with 1 marker | 6 | 1 | 1 |
| Scaffolds with 2 markers | 3 | 3 | 0 |
| Scaffolds with 3 markers | 3 | 2 | 0 |
| Scaffolds with >=4 markers | 23 | 23 | 0 |
| Total bases | 268,892,684 (90.4%) | 262,424,878 (88.2%) | 28,607,359 (9.6%) |

**Table S3. Repeat statistics of *Cucumis hystrix* genome**

| Repeat Type | Number | Total length(bp) | Percentage (%) |
| --- | --- | --- | --- |
| Satellites | 690 | 101,283 | 0.03 |
| Simple Repeats | 101,291 | 4,144,953 | 1.43 |
| Low Complexity | 24,433 | 1,223,842 | 0.44 |
| SINEs | 2,404 | 345,864 | 0.42 |
| LINEs | 13,923 | 6,186,718 | 2.13 |
| LTRs | 80,320 | 56,963,022 | 19.64 |
| DNA Transposons | 46,584 | 17,827,923 | 6.15 |
| Unclassified | 191,504 | 54,480,534 | 18.79 |

**Table S4. Statistics of genome assembly of *Cucumis* species**

|  | *C. sativus* | *C. hystrix* | *C. melo* |
| --- | --- | --- | --- |
| Karyotype | 2n=2x=14 | 2n=2x=24 | 2n=2x=24 |
| Estimated genome size (Mb) | 367 | 418 | 450 |
| Assembled genome size (Mb) | 226 | 298 | 417 |
| Scaffold N50 (Mb) | 31.1 | 14.1 | 4.68 |
| Contig N50 (kb) | 8,938 | 221 | 23 |
| Total gene number | 24,317 | 23,864 | 29,980 |

**Table S5. BUSCO analysis of genome assemblies and proteomes of several** **Cucurbitaceae species**

|  | Species | Complete (%) | Fragmented (%) | Missing (%) |
| --- | --- | --- | --- | --- |
| Genome | *Cucumis sativus* | 94.4 | 0.7 | 4.9 |
|  | *Cucumis hystrix* | 93.5 | 0.9 | 5.6 |
|  | *Cucumis melo* | 93.6 | 1.4 | 5.0 |
|  | *Citrullus lanatus* | 93.4 | 0.9 | 5.7 |
|  | *Lagenaria siceraria* | 94.0 | 0.8 | 5.2 |
|  | *Luffa cylindrica* | 94.8 | 1.0 | 4.2 |
|  | *Benincasa hispida* | 94.8 | 1.0 | 4.2 |
|  | *Momordica charantia* | 95.3 | 0.9 | 3.8 |
| Protein | *Cucumis sativus* | 95.7 | 2.0 | 2.3 |
|  | *Cucumis hystrix* | 94.5 | 2.4 | 3.1 |
|  | *Cucumis melo* | 91.2 | 5.0 | 3.8 |
|  | *Citrullus lanatus* | 94.4 | 2.1 | 3.5 |
|  | *Lagenaria siceraria* | 87.9 | 6.2 | 5.9 |
|  | *Luffa cylindrica* | 86.7 | 7.0 | 6.3 |
|  | *Benincasa hispida* | 93.8 | 1.9 | 4.3 |
|  | *Momordica charantia* | 95.1 | 1.1 | 3.8 |

**Table S6. Statistics of blocks of the five compared Cucurbitaceae speceies**

|  | total RBH number | total block number | number of RBH genes in blocks | number of RBH genes not in blocks | average RBH number of blocks |
| --- | --- | --- | --- | --- | --- |
| Csa-Chy | 16,916 | 119 | 16,246 | 670 | 137 |
| Csa-Cme | 16,131 | 240 | 15,333 | 798 | 64 |
| Chy-Cme | 15,200 | 182 | 14,399 | 801 | 79 |

Csa, *Cucumis Sativus*; Chy, *Cucumis hystrix*; Cme, *Cucumis melo*.

**Table S7. Detailed information of the syntenic blocks between *Cucumis hystrix* and cucumber**

| Chromosome ID of  *C. hystrix* | Start position | End position | Chromosome ID of cucumber | Start position | End position | Total orthologous gene number of the block |
| --- | --- | --- | --- | --- | --- | --- |
| chrH01 | 34538 | 6109966 | chr7 | 5818443 | 89991 | 589 |
| chrH01 | 6149604 | 12444599 | chr7 | 12696763 | 8361547 | 183 |
| chrH01 | 11609620 | 11919411 | chr7 | 9169521 | 9594454 | 9 |
| chrH01 | 12514929 | 12965962 | chr7 | 12726259 | 12981823 | 19 |
| chrH01 | 13038603 | 13360895 | chr7 | 6383159 | 6044837 | 11 |
| chrH01 | 13464070 | 13608265 | chr7 | 7790471 | 8243027 | 7 |
| chrH01 | 13645299 | 27086546 | chr7 | 13031810 | 22349737 | 798 |
| chrH01 | 26930317 | 27076690 | chr7 | 22434049 | 22358413 | 8 |
| chrH02 | 46761 | 7725040 | chr6 | 31036146 | 23934821 | 891 |
| chrH02 | 7735183 | 19934784 | chr1 | 24717776 | 31419088 | 443 |
| chrH02 | 20010828 | 20229320 | chr1 | 13997074 | 14171167 | 7 |
| chrH02 | 20359600 | 20529749 | chr1 | 12571844 | 12825966 | 6 |
| chrH02 | 20706619 | 20992873 | chr1 | 31653336 | 31491132 | 15 |
| chrH02 | 21215872 | 21579830 | chr1 | 13906779 | 13565690 | 13 |
| chrH02 | 21702989 | 23187264 | chr1 | 17373336 | 18372931 | 21 |
| chrH02 | 21955547 | 22433893 | chr1 | 17803087 | 17415098 | 12 |
| chrH02 | 23218653 | 24222749 | chr1 | 16405108 | 17221765 | 39 |
| chrH02 | 24306131 | 26019746 | chr1 | 14427007 | 16352086 | 114 |
| chrH02 | 26038714 | 26902868 | chr1 | 18396146 | 19192831 | 81 |
| chrH02 | 26916852 | 29947510 | chr1 | 2921174 | 23169 | 353 |
| chrH03 | 58102 | 1115057 | chr2 | 21810602 | 22654328 | 90 |
| chrH03 | 1134192 | 7312050 | chr6 | 729426 | 4583158 | 305 |
| chrH03 | 7694517 | 8521194 | chr6 | 17581258 | 17131921 | 28 |
| chrH03 | 8183746 | 11853566 | chr6 | 17346357 | 19868057 | 181 |
| chrH03 | 12301491 | 12506805 | chr2 | 21793830 | 21628423 | 18 |
| chrH03 | 12570502 | 12697538 | chr2 | 16015620 | 16124497 | 8 |
| chrH03 | 12785892 | 13566280 | chr6 | 20515139 | 19891028 | 61 |
| chrH03 | 13611147 | 13818182 | chr1 | 13448932 | 13242803 | 17 |
| chrH03 | 13822648 | 14026299 | chr2 | 16815488 | 17023711 | 29 |
| chrH03 | 14052448 | 18852970 | chr2 | 21611057 | 17038813 | 566 |
| chrH04 | 32351 | 8232607 | chr3 | 40864651 | 35357332 | 523 |
| chrH04 | 8353704 | 13126658 | chr3 | 27059627 | 24960393 | 121 |
| chrH04 | 13254111 | 16858353 | chr3 | 21932553 | 19207664 | 99 |
| chrH04 | 16957569 | 17496390 | chr3 | 24599070 | 24931900 | 26 |
| chrH04 | 17527760 | 18078933 | chr3 | 21950706 | 22640853 | 15 |
| chrH04 | 17737258 | 18069920 | chr3 | 22946665 | 22647416 | 14 |
| chrH04 | 18104154 | 18525646 | chr3 | 27444539 | 27084455 | 24 |
| chrH04 | 18357423 | 20110277 | chr3 | 27243080 | 28432093 | 76 |
| chrH04 | 20180571 | 27691730 | chr3 | 35334475 | 28456553 | 781 |
| chrH05 | 8975 | 270872 | chr4 | 11045189 | 10806895 | 32 |
| chrH05 | 296164 | 1054396 | chr4 | 13501922 | 14206583 | 77 |
| chrH05 | 1067282 | 2139541 | chr6 | 22609153 | 21572667 | 93 |
| chrH05 | 2204871 | 4606787 | chr2 | 9429130 | 11482127 | 124 |
| chrH05 | 4630550 | 5811124 | chr6 | 20543872 | 21564859 | 95 |
| chrH05 | 5834527 | 7261596 | chr2 | 8649342 | 9391724 | 32 |
| chrH05 | 7427205 | 8134523 | chr2 | 8128389 | 8635150 | 34 |
| chrH05 | 8223521 | 18561664 | chr2 | 8051055 | 160542 | 637 |
| chrH06 | 26902 | 10817974 | chr3 | 6373962 | 15768062 | 960 |
| chrH06 | 9286555 | 9468073 | chr3 | 14664150 | 14538513 | 11 |
| chrH06 | 11127980 | 11309062 | chr3 | 24346324 | 24520196 | 11 |
| chrH06 | 11494598 | 15384207 | chr3 | 19137226 | 15875682 | 112 |
| chrH06 | 15441873 | 17890550 | chr3 | 24306219 | 22509353 | 83 |
| chrH06 | 17913797 | 26981966 | chr3 | 6353996 | 150290 | 521 |
| chrH07 | 52536 | 3446184 | chr4 | 23246133 | 26459056 | 379 |
| chrH07 | 3631894 | 4761117 | chr4 | 23224740 | 22443714 | 68 |
| chrH07 | 5000249 | 5084463 | chr4 | 26577607 | 26502846 | 8 |
| chrH07 | 5304355 | 7250326 | chr4 | 17344455 | 18332380 | 70 |
| chrH07 | 7392126 | 21110492 | chr4 | 8296241 | 29096 | 722 |
| chrH07 | 14902801 | 15013655 | chr4 | 5421882 | 5503290 | 8 |
| chrH07 | 17661760 | 17791940 | scaffold78 | 7240 | 130492 | 7 |
| chrH07 | 21135637 | 21314851 | chr4 | 10054005 | 10238169 | 19 |
| chrH08 | 17229 | 2485985 | chr4 | 21245606 | 22345130 | 49 |
| chrH08 | 2552131 | 3433727 | chr4 | 16876044 | 17261008 | 17 |
| chrH08 | 3619701 | 4030873 | chr4 | 8489905 | 8337687 | 9 |
| chrH08 | 4209661 | 10316120 | chr4 | 18350606 | 21227099 | 129 |
| chrH08 | 10512073 | 12920631 | chr4 | 8516371 | 9990823 | 72 |
| chrH08 | 12941930 | 15733444 | chr4 | 16625684 | 14221274 | 205 |
| chrH08 | 13799934 | 13885979 | chr4 | 15836763 | 15911358 | 9 |
| chrH08 | 15747362 | 17122984 | chr6 | 22617915 | 23919674 | 146 |
| chrH08 | 17148303 | 18251100 | chr1 | 24711641 | 23770470 | 97 |
| chrH09 | 46298 | 2903414 | chr5 | 31883083 | 29525795 | 259 |
| chrH09 | 2909191 | 3607204 | chr5 | 22373833 | 21766640 | 68 |
| chrH09 | 3635399 | 4181050 | chr5 | 14631195 | 13789731 | 43 |
| chrH09 | 4290987 | 7807525 | chr5 | 19063465 | 16975806 | 69 |
| chrH09 | 7835232 | 10724594 | chr5 | 20033129 | 21746456 | 98 |
| chrH09 | 10767003 | 11848676 | chr5 | 23076363 | 24099252 | 82 |
| chrH09 | 11866495 | 16187987 | chr5 | 3952740 | 24049 | 451 |
| chrH10 | 49152 | 6593336 | chr5 | 7497031 | 12216558 | 105 |
| chrH10 | 6703263 | 13039558 | chr5 | 7428715 | 3964141 | 236 |
| chrH10 | 13037291 | 18135595 | chr5 | 24111438 | 29124459 | 612 |
| chrH11 | 44596 | 226146 | chr2 | 15672282 | 15825047 | 13 |
| chrH11 | 146445 | 4555116 | chr2 | 15751361 | 11635777 | 323 |
| chrH11 | 4611704 | 8744270 | chr2 | 24790132 | 22662708 | 154 |
| chrH11 | 8774512 | 11377040 | chr6 | 7507656 | 8511621 | 59 |
| chrH11 | 11540486 | 14176459 | chr6 | 10034670 | 11835763 | 140 |
| chrH11 | 14240634 | 15177338 | chr6 | 10023959 | 9373256 | 46 |
| chrH11 | 15321794 | 17924086 | chr6 | 8532920 | 9365243 | 68 |
| chrH11 | 18034766 | 18621615 | chr6 | 12294556 | 12696496 | 42 |
| chrH11 | 18663089 | 18991117 | chr6 | 14950404 | 15208872 | 24 |
| chrH11 | 18915496 | 19419834 | chr6 | 15115860 | 14631663 | 12 |
| chrH11 | 19308852 | 19552246 | chr6 | 14516459 | 14750281 | 22 |
| chrH11 | 19646234 | 20167374 | chr6 | 12274814 | 11888239 | 38 |
| chrH11 | 20181060 | 21148461 | chr6 | 13986064 | 13104775 | 29 |
| chrH11 | 21186251 | 21460690 | chr6 | 4669439 | 4924854 | 29 |
| chrH11 | 21502510 | 21727427 | chr6 | 12923716 | 12754947 | 22 |
| chrH11 | 21809313 | 22191947 | chr6 | 15901140 | 15295937 | 43 |
| chrH11 | 22281875 | 22442848 | chr6 | 16877963 | 16734772 | 12 |
| chrH11 | 22535569 | 23317621 | chr6 | 25281 | 714471 | 77 |
| chrH11 | 23333606 | 26034284 | chr6 | 7482700 | 4938500 | 252 |
| chrH11 | 23823342 | 24070793 | chr6 | 6810066 | 7039659 | 21 |
| chrH12 | 46043 | 5807623 | chr1 | 19722369 | 23752962 | 261 |
| chrH12 | 4389361 | 4998920 | chr1 | 23363435 | 22962003 | 28 |
| chrH12 | 5846051 | 7665973 | chr1 | 32775308 | 31689067 | 58 |
| chrH12 | 7689552 | 19553591 | chr1 | 12092251 | 3151984 | 779 |
| scaffold25_size3045174 | 26572 | 787678 | chr5 | 19262921 | 20012310 | 36 |
| scaffold25_size3045174 | 845621 | 1374577 | chr5 | 16684368 | 15744409 | 21 |
| scaffold25_size3045174 | 1383769 | 2313971 | chr5 | 14684040 | 15705558 | 28 |
| scaffold25_size3045174 | 2736664 | 2854206 | chr5 | 13779575 | 13672035 | 7 |
| scaffold25_size3045174 | 2922881 | 3014339 | chr5 | 22952897 | 23035115 | 7 |
| scaffold26_size3936274 | 231886 | 2598689 | chr5 | 12327743 | 13373576 | 30 |
| scaffold26_size3936274 | 2805913 | 3630100 | chr5 | 22852907 | 22400034 | 13 |
| scaffold32_size1584725 | 89440 | 438121 | chr4 | 16853823 | 16664339 | 7 |
| scaffold32_size1584725 | 684556 | 934216 | chr4 | 12972807 | 13310965 | 7 |
| scaffold34_size1410443 | 290387 | 666800 | chr1 | 3130456 | 2927121 | 11 |
| scaffold34_size1410443 | 715718 | 1396395 | chr1 | 19261836 | 19700956 | 28 |
| scaffold36_size1158972 | 71290 | 1016963 | chr5 | 29377965 | 29180844 | 7 |
| scaffold38_size900072 | 77325 | 805088 | chr5 | 10251470 | 10521038 | 9 |
| scaffold39_size246216 | 26759 | 218171 | scaffold18_1 | 518997 | 676974 | 6 |
| scaffold75_size41661 | 11455 | 38478 | chr3 | 9450807 | 9429559 | 8 |

**Table S8. Detailed information of the syntenic blocks between *Cucumis hystrix* and melon**

| Chromosome ID of *C. hystrix* | Start position | End position | Chromosome ID of melon | Start position | End position | Total orthologous gene number of the block |
| --- | --- | --- | --- | --- | --- | --- |
| chrH01 | 12787 | 3984892 | chr1 | 13781 | 4537278 | 384 |
| chrH01 | 3990542 | 4155821 | chr1 | 27104679 | 27345583 | 18 |
| chrH01 | 4151266 | 9214524 | chr1 | 14748789 | 22040062 | 210 |
| chrH01 | 9451967 | 11395405 | chr1 | 12274088 | 14542747 | 42 |
| chrH01 | 11507833 | 12144529 | chr1 | 12219455 | 11411276 | 19 |
| chrH01 | 12175741 | 12444599 | chr1 | 9916087 | 10321740 | 13 |
| chrH01 | 12514929 | 12776919 | chr1 | 9306376 | 9678622 | 8 |
| chrH01 | 12819350 | 13608265 | chr1 | 11362264 | 10360871 | 29 |
| chrH01 | 13691512 | 15538006 | chr1 | 7253995 | 9264534 | 22 |
| chrH01 | 13840263 | 14072020 | chr1 | 7228655 | 7007012 | 7 |
| chrH01 | 15550977 | 15858739 | chr1 | 6345127 | 6653184 | 9 |
| chrH01 | 15657053 | 17809707 | chr1 | 6428415 | 4678226 | 30 |
| chrH01 | 17867633 | 18197657 | chr1 | 24094236 | 23188514 | 8 |
| chrH01 | 17966564 | 20199932 | chr1 | 23953098 | 27045354 | 43 |
| chrH01 | 20226930 | 20593368 | chr1 | 22395204 | 22093236 | 12 |
| chrH01 | 20261563 | 20551746 | chr1 | 22367847 | 22989428 | 10 |
| chrH01 | 20614277 | 22181668 | chr1 | 27448769 | 29984444 | 58 |
| chrH01 | 21050761 | 21254202 | chr1 | 28466027 | 28161328 | 14 |
| chrH01 | 21958074 | 22311408 | chr1 | 30230274 | 29729908 | 33 |
| chrH01 | 22430768 | 23401023 | chr1 | 31600961 | 30340751 | 100 |
| chrH01 | 23422238 | 27041364 | chr1 | 31674796 | 35298696 | 358 |
| chrH01 | 26910687 | 27086546 | chr1 | 35380903 | 35252339 | 9 |
| chrH02 | 46761 | 2883298 | chr8 | 44902 | 2859316 | 342 |
| chrH02 | 2885619 | 6799567 | chr8 | 6754971 | 2865747 | 431 |
| chrH02 | 6807904 | 7725040 | chr8 | 6765372 | 7844061 | 45 |
| chrH02 | 7735183 | 14745205 | chr2 | 964587 | 8728084 | 309 |
| chrH02 | 14772819 | 18983417 | chr2 | 10399274 | 14171221 | 54 |
| chrH02 | 17335737 | 18042955 | chr2 | 13376840 | 12682383 | 8 |
| chrH02 | 19331828 | 19951445 | chr2 | 19560256 | 20045478 | 11 |
| chrH02 | 19331828 | 20229320 | chr2 | 19560256 | 19104048 | 8 |
| chrH02 | 20359600 | 21126026 | chr2 | 20615324 | 20080743 | 18 |
| chrH02 | 21191168 | 25984232 | chr2 | 19249652 | 14226860 | 159 |
| chrH02 | 22013364 | 22433893 | chr2 | 18434250 | 18743740 | 9 |
| chrH02 | 25505352 | 25544802 | chr2 | 14766661 | 14817381 | 6 |
| chrH02 | 25990611 | 29936559 | chr2 | 21400037 | 26175612 | 367 |
| chrH02 | 27760949 | 27978210 | chr2 | 24120370 | 23898017 | 23 |
| chrH02 | 28978234 | 29086447 | chr2 | 25309293 | 25155656 | 6 |
| chrH02 | 29465472 | 29577979 | chr2 | 25799578 | 25695599 | 11 |
| chrH03 | 58102 | 1920946 | chr3 | 10207 | 3724479 | 161 |
| chrH03 | 1930888 | 2232727 | chr3 | 19939402 | 19130031 | 22 |
| chrH03 | 2253478 | 3291753 | chr3 | 16247883 | 18253454 | 31 |
| chrH03 | 3011392 | 3379770 | chr3 | 18873678 | 17950905 | 8 |
| chrH03 | 3394668 | 6047222 | chr3 | 16149842 | 10184311 | 52 |
| chrH03 | 6127900 | 6798901 | chr3 | 21432643 | 19978379 | 51 |
| chrH03 | 6838838 | 7657385 | chr3 | 3793863 | 5328182 | 25 |
| chrH03 | 7664683 | 8498126 | chr3 | 6646495 | 5418366 | 22 |
| chrH03 | 8543738 | 9778650 | chr3 | 22851205 | 23998578 | 94 |
| chrH03 | 9848125 | 11525346 | chr3 | 6796691 | 9351866 | 33 |
| chrH03 | 11248639 | 11582934 | chr3 | 9653364 | 9300307 | 6 |
| chrH03 | 11630945 | 11853566 | chr3 | 24221537 | 24022778 | 16 |
| chrH03 | 12301491 | 13566280 | chr3 | 21441537 | 22845260 | 78 |
| chrH03 | 13627638 | 18852970 | chr3 | 24238670 | 29372309 | 573 |
| chrH04 | 13616 | 6896261 | chr4 | 171412 | 9133637 | 422 |
| chrH04 | 7092137 | 7775120 | chr4 | 16190877 | 17203337 | 19 |
| chrH04 | 7483397 | 7711737 | chr4 | 17683214 | 17241068 | 12 |
| chrH04 | 7560310 | 8676397 | chr4 | 17469805 | 18816411 | 19 |
| chrH04 | 8760282 | 9958556 | chr4 | 20291547 | 18887795 | 34 |
| chrH04 | 9941610 | 11035051 | chr4 | 22259734 | 20422235 | 36 |
| chrH04 | 10984377 | 11426584 | chr4 | 23028887 | 21834793 | 8 |
| chrH04 | 11445231 | 13126658 | chr4 | 11362405 | 12654647 | 16 |
| chrH04 | 13274074 | 13930519 | chr4 | 15312976 | 16014658 | 27 |
| chrH04 | 14145338 | 15446521 | chr4 | 11302117 | 9414758 | 30 |
| chrH04 | 15596855 | 17496390 | chr4 | 14399474 | 12801207 | 41 |
| chrH04 | 17536815 | 18288698 | chr4 | 15258576 | 14470559 | 36 |
| chrH04 | 18349679 | 27691730 | chr4 | 23366111 | 33108545 | 761 |
| chrH04 | 20482931 | 20604129 | chr4 | 25773715 | 25660675 | 8 |
| chrH04 | 25733165 | 25803494 | chr4 | 31213182 | 31143355 | 9 |
| chrH05 | 8975 | 2139541 | chr5 | 9506 | 2212824 | 189 |
| chrH05 | 2255453 | 3857698 | chr5 | 7360919 | 4919855 | 68 |
| chrH05 | 3892812 | 4819143 | chr5 | 4564811 | 3442287 | 52 |
| chrH05 | 4859293 | 5517994 | chr5 | 2664789 | 3396744 | 44 |
| chrH05 | 5535620 | 5796060 | chr5 | 2536303 | 2232932 | 25 |
| chrH05 | 5834527 | 10556183 | chr5 | 7950688 | 16199342 | 94 |
| chrH05 | 9686065 | 10212014 | chr5 | 17013085 | 16276844 | 6 |
| chrH05 | 9956925 | 18582335 | chr5 | 16576462 | 28323598 | 480 |
| chrH05 | 16984422 | 17241305 | chr5 | 26945840 | 26672224 | 27 |
| chrH06 | 26902 | 7297700 | chr6 | 7842 | 7647601 | 732 |
| chrH06 | 7133941 | 7230880 | chr6 | 7592256 | 7497902 | 10 |
| chrH06 | 7258414 | 7325337 | chr6 | 7691212 | 7606811 | 8 |
| chrH06 | 7372772 | 11135953 | chr6 | 13471241 | 8477504 | 129 |
| chrH06 | 11152121 | 11255627 | chr3 | 7358381 | 7250253 | 6 |
| chrH06 | 11309062 | 11592713 | chr6 | 8009957 | 8414910 | 9 |
| chrH06 | 11614021 | 11776157 | chr6 | 7942654 | 7723706 | 12 |
| chrH06 | 11850392 | 21269597 | chr6 | 13266443 | 28844538 | 230 |
| chrH06 | 15768380 | 16099859 | chr6 | 20506391 | 19759392 | 12 |
| chrH06 | 21214802 | 22022201 | chr6 | 30319589 | 29070927 | 32 |
| chrH06 | 22068378 | 26981966 | chr6 | 30438157 | 35909218 | 342 |
| chrH07 | 84221 | 585051 | chr7 | 4381064 | 5253327 | 49 |
| chrH07 | 607087 | 3446184 | chr7 | 2844521 | 32019 | 307 |
| chrH07 | 3637346 | 3764773 | chr7 | 4351583 | 4171416 | 7 |
| chrH07 | 3772206 | 3839388 | chr7 | 2923056 | 2850456 | 8 |
| chrH07 | 3842344 | 9725374 | chr7 | 5273442 | 13212158 | 120 |
| chrH07 | 4084846 | 4428568 | chr7 | 6094019 | 5827421 | 14 |
| chrH07 | 4527053 | 4670170 | chr7 | 6557699 | 6391518 | 6 |
| chrH07 | 4677800 | 4761117 | chr7 | 6733082 | 6622531 | 7 |
| chrH07 | 8961275 | 10827112 | chr7 | 16990660 | 18238232 | 17 |
| chrH07 | 10950796 | 11998107 | chr7 | 15560483 | 16497770 | 16 |
| chrH07 | 12114208 | 12896164 | chr7 | 14305307 | 15252435 | 7 |
| chrH07 | 12267094 | 13091344 | chr7 | 15318608 | 13641774 | 14 |
| chrH07 | 13443835 | 21311777 | chr7 | 18375538 | 26755586 | 552 |
| chrH07 | 15739682 | 16027936 | chr7 | 21087443 | 20689737 | 25 |
| chrH07 | 16349861 | 16529832 | chr7 | 21783374 | 21563613 | 17 |
| chrH08 | 17229 | 1374865 | chr8 | 20019314 | 18614539 | 14 |
| chrH08 | 803057 | 1738007 | chr8 | 18264801 | 17670364 | 6 |
| chrH08 | 1863138 | 2446520 | chr8 | 23461755 | 23103283 | 11 |
| chrH08 | 2654037 | 3278028 | chr8 | 29068102 | 28107181 | 10 |
| chrH08 | 4158194 | 6751819 | chr8 | 14326223 | 10596733 | 50 |
| chrH08 | 6858248 | 7493492 | chr8 | 31678483 | 32350913 | 15 |
| chrH08 | 7676568 | 10316120 | chr8 | 23070558 | 20040338 | 43 |
| chrH08 | 10685572 | 11350503 | chr8 | 15715334 | 14670069 | 15 |
| chrH08 | 11614374 | 14205594 | chr8 | 23537540 | 27634037 | 116 |
| chrH08 | 14324133 | 15672417 | chr8 | 31350538 | 29556861 | 120 |
| chrH08 | 15700071 | 15846002 | chr5 | 4731882 | 4888072 | 22 |
| chrH08 | 15883568 | 15999764 | chr8 | 29502871 | 29377383 | 11 |
| chrH08 | 16043729 | 17111674 | chr8 | 9177194 | 7869291 | 102 |
| chrH08 | 17148303 | 18099197 | chr2 | 950903 | 171238 | 68 |
| chrH08 | 17932831 | 18233216 | chr2 | 10574 | 291923 | 22 |
| chrH09 | 46298 | 3607204 | chr9 | 31143 | 4710356 | 277 |
| chrH09 | 1236603 | 1416564 | chr9 | 1437853 | 1316421 | 13 |
| chrH09 | 3635399 | 4181050 | chr9 | 16992519 | 17754138 | 39 |
| chrH09 | 4290987 | 10724594 | chr9 | 13900457 | 4753747 | 142 |
| chrH09 | 8036342 | 8199635 | chr9 | 8742531 | 9113197 | 6 |
| chrH09 | 10767003 | 10980872 | chr9 | 18459586 | 18313499 | 7 |
| chrH09 | 10801924 | 16187987 | chr9 | 18426978 | 24102483 | 456 |
| chrH09 | 10878012 | 11006838 | chr9 | 18875040 | 18356060 | 8 |
| chrH09 | 12811733 | 12954146 | chr9 | 20828221 | 20674614 | 19 |
| chrH10 | 17815 | 1467766 | chr10 | 14423994 | 16804461 | 19 |
| chrH10 | 1618362 | 2732321 | chr10 | 10483142 | 11973855 | 14 |
| chrH10 | 3921092 | 4181588 | chr10 | 22104048 | 21844814 | 6 |
| chrH10 | 5194155 | 5475011 | chr7 | 3284930 | 2967237 | 8 |
| chrH10 | 5497061 | 6040521 | chr10 | 21054257 | 21341259 | 9 |
| chrH10 | 6106520 | 6593336 | chr2 | 8977003 | 9703519 | 11 |
| chrH10 | 6703263 | 8786252 | chr10 | 8402288 | 10245599 | 41 |
| chrH10 | 8833335 | 9603860 | chr10 | 16884600 | 17800261 | 19 |
| chrH10 | 9711476 | 10765480 | chr10 | 13703443 | 12235552 | 32 |
| chrH10 | 10886265 | 18135595 | chr10 | 8150796 | 14913 | 648 |
| chrH10 | 14108079 | 14145780 | chr10 | 3983518 | 4020332 | 7 |
| chrH11 | 44596 | 899297 | chr11 | 10405 | 903041 | 56 |
| chrH11 | 931227 | 2868637 | chr11 | 2300094 | 4327474 | 132 |
| chrH11 | 1551491 | 1886431 | chr11 | 3267621 | 2888608 | 19 |
| chrH11 | 2957176 | 3561963 | chr11 | 2067812 | 952715 | 42 |
| chrH11 | 3636860 | 3756694 | chr11 | 2273193 | 2149730 | 7 |
| chrH11 | 3902307 | 11308687 | chr11 | 4471334 | 16320534 | 199 |
| chrH11 | 4555116 | 5171981 | chr11 | 6472580 | 5626901 | 16 |
| chrH11 | 11540486 | 12541018 | chr11 | 25810538 | 26771987 | 75 |
| chrH11 | 11776652 | 11902679 | chr0 | 844105 | 673583 | 14 |
| chrH11 | 12822550 | 15177338 | chr11 | 24222445 | 21497421 | 69 |
| chrH11 | 15321794 | 15839033 | chr11 | 16122391 | 15789628 | 11 |
| chrH11 | 15979497 | 17614589 | chr11 | 19408636 | 17911028 | 26 |
| chrH11 | 17444435 | 17648559 | chr11 | 17708403 | 18000981 | 6 |
| chrH11 | 17742246 | 17924086 | chr11 | 21106053 | 21477723 | 12 |
| chrH11 | 18037261 | 18903518 | chr11 | 20536102 | 19521315 | 45 |
| chrH11 | 18663089 | 18751173 | chr11 | 25313096 | 25179061 | 9 |
| chrH11 | 18826616 | 19000623 | chr11 | 19461130 | 19628786 | 18 |
| chrH11 | 19055751 | 19552246 | chr11 | 25348817 | 25805445 | 29 |
| chrH11 | 19646234 | 19854002 | chr11 | 20562494 | 20804160 | 16 |
| chrH11 | 19986708 | 20102261 | chr0 | 132289 | 385540 | 11 |
| chrH11 | 20112541 | 20960879 | chr11 | 25068544 | 24351332 | 8 |
| chrH11 | 20352877 | 20409668 | chr11 | 24806323 | 24896219 | 6 |
| chrH11 | 21047281 | 26034284 | chr11 | 26780117 | 31425741 | 431 |
| chrH12 | 46043 | 1214690 | chr12 | 20582524 | 21314331 | 63 |
| chrH12 | 1331450 | 3667210 | chr12 | 3103578 | 250983 | 135 |
| chrH12 | 3723400 | 5878030 | chr12 | 20024839 | 17253113 | 65 |
| chrH12 | 5891874 | 6506921 | chr12 | 17241169 | 17756115 | 7 |
| chrH12 | 6198948 | 6607420 | chr12 | 16903530 | 17643494 | 10 |
| chrH12 | 6663577 | 8093927 | chr12 | 16881186 | 15500165 | 41 |
| chrH12 | 8132996 | 8591882 | chr12 | 13573588 | 12792365 | 23 |
| chrH12 | 8608375 | 9389090 | chr12 | 14200789 | 15332176 | 10 |
| chrH12 | 8941543 | 9336968 | chr12 | 15172310 | 14705812 | 13 |
| chrH12 | 9104936 | 9784804 | chr12 | 13759047 | 14147675 | 12 |
| chrH12 | 9753544 | 14328026 | chr12 | 12735700 | 3455107 | 120 |
| chrH12 | 14384417 | 19509744 | chr12 | 21320143 | 26255697 | 504 |
| chrH12 | 19349579 | 19566663 | chr12 | 26396474 | 26194795 | 10 |
| scaffold25_size3045174 | 26572 | 2607715 | chr9 | 16879094 | 14564175 | 65 |
| scaffold25_size3045174 | 1869404 | 2648362 | chr9 | 14056950 | 14496580 | 12 |
| scaffold25_size3045174 | 2736664 | 3014339 | chr9 | 17833249 | 18113578 | 13 |
| scaffold26_size3936274 | 489116 | 1254277 | chr10 | 20800790 | 19780499 | 6 |
| scaffold26_size3936274 | 2136301 | 3630100 | chr10 | 23243704 | 24534204 | 10 |
| scaffold32_size1584725 | 395666 | 834857 | chr8 | 28574367 | 29315398 | 6 |
| scaffold32_size1584725 | 861587 | 1485204 | chr8 | 9884755 | 9196631 | 7 |
| scaffold34_size1410443 | 583863 | 1396395 | chr12 | 20076944 | 20572059 | 29 |
| scaffold38_size900072 | 57613 | 717507 | chr10 | 18741548 | 19325449 | 8 |

**Table S9. Detailed information of the syntenic blocks between cucumber and melon**

| Chromosome ID of cucumber | Start position | End position | Chromosome ID of melon | Start position | End position | Total orthologous gene number of the block |
| --- | --- | --- | --- | --- | --- | --- |
| chr1 | 29387 | 1914711 | chr2 | 26175612 | 24159490 | 217 |
| chr1 | 384375 | 488576 | chr2 | 25695599 | 25799578 | 12 |
| chr1 | 874726 | 983878 | chr2 | 25155656 | 25314589 | 8 |
| chr1 | 1926707 | 2138595 | chr2 | 23898017 | 24120370 | 27 |
| chr1 | 2145973 | 2921174 | chr2 | 23892054 | 22880269 | 90 |
| chr1 | 2927121 | 2980830 | chr12 | 20168851 | 20076944 | 6 |
| chr1 | 2990008 | 3130456 | chr12 | 218118 | 126533 | 8 |
| chr1 | 3151984 | 3327797 | chr12 | 26206674 | 26396474 | 10 |
| chr1 | 3333214 | 7940355 | chr12 | 26188486 | 21320143 | 550 |
| chr1 | 7972816 | 10685647 | chr12 | 3417033 | 12735700 | 129 |
| chr1 | 10668648 | 10798859 | chr12 | 14147675 | 13876631 | 9 |
| chr1 | 10707463 | 11098761 | chr12 | 14113882 | 15172310 | 14 |
| chr1 | 10857878 | 11269382 | chr12 | 15332176 | 14200789 | 10 |
| chr1 | 11289304 | 11750628 | chr12 | 12792365 | 13573588 | 18 |
| chr1 | 11760990 | 12092251 | chr12 | 15500165 | 15810914 | 13 |
| chr1 | 12571844 | 12825966 | chr2 | 20615324 | 20554856 | 7 |
| chr1 | 13242803 | 13419139 | chr3 | 24434493 | 24238670 | 15 |
| chr1 | 13623567 | 14171167 | chr2 | 19325478 | 19104048 | 8 |
| chr1 | 13655392 | 13987047 | chr2 | 18767672 | 19247248 | 11 |
| chr1 | 14427007 | 16316433 | chr2 | 16239161 | 14226860 | 95 |
| chr1 | 15831391 | 15863204 | chr2 | 14766661 | 14817381 | 6 |
| chr1 | 16405108 | 17186876 | chr2 | 17494107 | 16341342 | 37 |
| chr1 | 17415098 | 18372931 | chr2 | 18743740 | 17530640 | 30 |
| chr1 | 18396146 | 19192831 | chr2 | 21503626 | 22866762 | 82 |
| chr1 | 19261836 | 20545800 | chr12 | 20183427 | 21314331 | 87 |
| chr1 | 20572122 | 22582839 | chr12 | 3287328 | 250983 | 143 |
| chr1 | 22621357 | 22940952 | chr12 | 20031494 | 19421576 | 19 |
| chr1 | 22962003 | 23363435 | chr12 | 18524147 | 19348386 | 29 |
| chr1 | 23372452 | 23752962 | chr12 | 18424817 | 17816476 | 20 |
| chr1 | 23786305 | 24029687 | chr2 | 291923 | 10574 | 24 |
| chr1 | 23895919 | 29117812 | chr2 | 180860 | 8728084 | 382 |
| chr1 | 29134510 | 30968363 | chr2 | 10399274 | 14031249 | 56 |
| chr1 | 30351437 | 30609458 | chr2 | 13297838 | 12682383 | 7 |
| chr1 | 31224681 | 31653336 | chr2 | 19560256 | 20424586 | 22 |
| chr1 | 31689067 | 32659877 | chr12 | 15832582 | 17423547 | 30 |
| chr1 | 32281075 | 32585445 | chr12 | 17643494 | 16903530 | 10 |
| chr1 | 32281075 | 32361552 | chr12 | 17643494 | 17756115 | 6 |
| chr1 | 32361552 | 32790665 | chr12 | 17756115 | 17253113 | 6 |
| chr2 | 183166 | 1385884 | chr5 | 28271677 | 26985588 | 146 |
| chr2 | 1390919 | 1638518 | chr5 | 26672224 | 26945840 | 28 |
| chr2 | 1650211 | 6931625 | chr5 | 26637776 | 16799139 | 360 |
| chr2 | 6009002 | 6065630 | chr5 | 18460286 | 18629729 | 6 |
| chr2 | 6491328 | 6665724 | chr5 | 16199342 | 14933535 | 6 |
| chr2 | 6531583 | 7053776 | chr5 | 16047338 | 17026353 | 13 |
| chr2 | 7077186 | 8051055 | chr5 | 14744264 | 11772956 | 33 |
| chr2 | 8128389 | 8635150 | chr5 | 9973270 | 11645832 | 32 |
| chr2 | 8649342 | 9391724 | chr5 | 7950688 | 9805790 | 29 |
| chr2 | 9402438 | 10799190 | chr5 | 7531331 | 4919855 | 78 |
| chr2 | 10825996 | 11482127 | chr5 | 4564811 | 3728018 | 43 |
| chr2 | 11666727 | 12077243 | chr11 | 5536266 | 4471334 | 36 |
| chr2 | 12157128 | 12252956 | chr11 | 2122625 | 2260118 | 10 |
| chr2 | 12261298 | 12779153 | chr11 | 952715 | 2067812 | 51 |
| chr2 | 12872517 | 14837227 | chr11 | 4327474 | 2300094 | 160 |
| chr2 | 14007912 | 14287574 | chr11 | 2888608 | 3267621 | 20 |
| chr2 | 14932431 | 15645442 | chr11 | 794043 | 196216 | 53 |
| chr2 | 15672282 | 15825047 | chr11 | 10405 | 176320 | 14 |
| chr2 | 16015620 | 16124497 | chr3 | 21937307 | 22055068 | 9 |
| chr2 | 16815488 | 17023711 | chr3 | 24438491 | 24626168 | 27 |
| chr2 | 17038813 | 21611057 | chr3 | 29372309 | 24645566 | 557 |
| chr2 | 21628423 | 21793830 | chr3 | 21882253 | 21441537 | 16 |
| chr2 | 21810602 | 22654328 | chr3 | 10207 | 1872980 | 91 |
| chr2 | 22662708 | 24808441 | chr11 | 12685787 | 5547216 | 119 |
| chr2 | 24520463 | 24790132 | chr11 | 5626901 | 6405895 | 19 |
| chr3 | 150290 | 4142428 | chr6 | 35909218 | 30438157 | 367 |
| chr3 | 4163670 | 4649811 | chr6 | 29070927 | 30319589 | 35 |
| chr3 | 4613663 | 6353996 | chr6 | 28844538 | 23374572 | 99 |
| chr3 | 5945423 | 6018099 | chr6 | 24631386 | 24876275 | 6 |
| chr3 | 6373962 | 13168566 | chr6 | 7842 | 7647601 | 788 |
| chr3 | 13028600 | 13104877 | chr6 | 7584259 | 7489594 | 10 |
| chr3 | 13121424 | 13194197 | chr6 | 7691212 | 7606811 | 8 |
| chr3 | 13231512 | 15768062 | chr6 | 13471241 | 8497408 | 124 |
| chr3 | 13311349 | 13374290 | chr6 | 13123909 | 13331289 | 6 |
| chr3 | 14538513 | 14664150 | chr6 | 10360302 | 10544789 | 9 |
| chr3 | 15875682 | 18848899 | chr6 | 18966203 | 13266443 | 72 |
| chr3 | 18862231 | 19108490 | chr6 | 7723706 | 8054458 | 11 |
| chr3 | 19043599 | 19122200 | chr6 | 8414910 | 8043239 | 7 |
| chr3 | 19207664 | 19992652 | chr4 | 13355615 | 14399474 | 21 |
| chr3 | 20063898 | 20917297 | chr4 | 9414758 | 11302117 | 31 |
| chr3 | 21122492 | 22640853 | chr4 | 16014658 | 14618807 | 45 |
| chr3 | 22509353 | 23849584 | chr6 | 23350151 | 20584586 | 60 |
| chr3 | 22640853 | 22923410 | chr4 | 14618807 | 14954380 | 14 |
| chr3 | 23866160 | 24102527 | chr6 | 19759392 | 20506391 | 13 |
| chr3 | 24365078 | 24468662 | chr3 | 7358381 | 7250253 | 6 |
| chr3 | 24635527 | 25417486 | chr4 | 13512296 | 11422156 | 37 |
| chr3 | 25432821 | 25711280 | chr4 | 21834793 | 23028887 | 8 |
| chr3 | 25745030 | 26373868 | chr4 | 20422235 | 22259734 | 38 |
| chr3 | 26355222 | 26857437 | chr4 | 18887795 | 20291547 | 34 |
| chr3 | 26944399 | 27059627 | chr4 | 18816411 | 18465585 | 9 |
| chr3 | 27084455 | 27232854 | chr4 | 23527253 | 23390492 | 9 |
| chr3 | 27250732 | 28429033 | chr4 | 23366111 | 25119843 | 66 |
| chr3 | 27291797 | 27444539 | chr4 | 14470559 | 14604410 | 11 |
| chr3 | 28456553 | 35334475 | chr4 | 33108545 | 25159625 | 748 |
| chr3 | 30277663 | 30338746 | chr4 | 31143355 | 31213182 | 10 |
| chr3 | 34981698 | 35083944 | chr4 | 25660675 | 25773715 | 10 |
| chr3 | 35359754 | 35680050 | chr4 | 18333018 | 17469805 | 7 |
| chr3 | 35522920 | 35725384 | chr4 | 17148226 | 17683214 | 14 |
| chr3 | 35562047 | 36017024 | chr4 | 17241068 | 16155432 | 21 |
| chr3 | 36065746 | 40864651 | chr4 | 9133637 | 182799 | 438 |
| chr3 | 36545417 | 36601229 | chr4 | 6692252 | 7225402 | 7 |
| chr4 | 29096 | 4320324 | chr7 | 26560798 | 21813921 | 435 |
| chr4 | 2049349 | 2100750 | chr7 | 24329573 | 24387269 | 8 |
| chr4 | 3397449 | 3447301 | chr7 | 22798494 | 22852349 | 6 |
| chr4 | 4327467 | 4494867 | chr7 | 21563613 | 21783374 | 19 |
| chr4 | 4516997 | 4750078 | chr7 | 21532250 | 21174825 | 23 |
| chr4 | 4761019 | 5016854 | chr7 | 20689737 | 21087443 | 26 |
| chr4 | 5039442 | 6213133 | chr7 | 20590637 | 18302354 | 74 |
| chr4 | 5421882 | 5503290 | chr7 | 19746993 | 19899446 | 7 |
| chr4 | 6018325 | 6197273 | chr7 | 16951170 | 16551496 | 7 |
| chr4 | 6318466 | 6969107 | chr7 | 13641774 | 15916697 | 16 |
| chr4 | 6419225 | 6660998 | chr7 | 15252435 | 14305307 | 6 |
| chr4 | 6728989 | 7051972 | chr7 | 16416799 | 15574860 | 13 |
| chr4 | 7122936 | 7749206 | chr7 | 18238232 | 17040255 | 18 |
| chr4 | 7542328 | 8194422 | chr7 | 13235784 | 10726103 | 27 |
| chr4 | 8414560 | 8915092 | chr8 | 16673369 | 14690883 | 21 |
| chr4 | 9197213 | 9990823 | chr8 | 23537540 | 25316488 | 49 |
| chr4 | 10054005 | 10242415 | chr7 | 26585909 | 26762401 | 18 |
| chr4 | 10806895 | 11045189 | chr5 | 313876 | 9506 | 30 |
| chr4 | 13501922 | 14206583 | chr5 | 345131 | 1105165 | 77 |
| chr4 | 14221274 | 14254017 | chr5 | 4765806 | 4726101 | 6 |
| chr4 | 14282490 | 15474328 | chr8 | 29556861 | 31350538 | 130 |
| chr4 | 15584187 | 16625684 | chr8 | 27634037 | 25336122 | 63 |
| chr4 | 15836763 | 15911358 | chr8 | 27044583 | 27191505 | 8 |
| chr4 | 16897584 | 17174442 | chr8 | 28885859 | 28107181 | 10 |
| chr4 | 17201808 | 17286697 | chr8 | 10500119 | 10107305 | 6 |
| chr4 | 17344455 | 18332380 | chr7 | 7678718 | 10234806 | 69 |
| chr4 | 18361348 | 19431056 | chr8 | 14237857 | 10596733 | 52 |
| chr4 | 19526264 | 19794363 | chr8 | 31704061 | 32350913 | 13 |
| chr4 | 19858339 | 22032660 | chr8 | 23070558 | 17670364 | 63 |
| chr4 | 22083111 | 22320397 | chr8 | 23461755 | 23103283 | 11 |
| chr4 | 22353939 | 23043191 | chr7 | 7037168 | 5273442 | 26 |
| chr4 | 22443714 | 22508350 | chr7 | 6622531 | 6715871 | 9 |
| chr4 | 22528212 | 22628201 | chr7 | 6391518 | 6557699 | 7 |
| chr4 | 22659819 | 22850063 | chr7 | 5827421 | 6094019 | 14 |
| chr4 | 23046395 | 23114019 | chr7 | 2850456 | 2923056 | 7 |
| chr4 | 23120734 | 23788622 | chr7 | 4171416 | 5253327 | 62 |
| chr4 | 23807254 | 26479651 | chr7 | 2844521 | 17780 | 330 |
| chr4 | 26502846 | 26577607 | chr7 | 7457706 | 7161584 | 8 |
| chr5 | 24049 | 3015015 | chr9 | 24102483 | 20852605 | 352 |
| chr5 | 3022835 | 3163322 | chr9 | 20674614 | 20828221 | 20 |
| chr5 | 3167896 | 3952740 | chr9 | 20667951 | 19748555 | 79 |
| chr5 | 3972636 | 5408732 | chr10 | 5163305 | 8150796 | 102 |
| chr5 | 5436285 | 6110491 | chr10 | 12235552 | 13703443 | 33 |
| chr5 | 6161258 | 6562937 | chr10 | 17800261 | 16884600 | 20 |
| chr5 | 6597000 | 7428715 | chr10 | 10242464 | 8402288 | 45 |
| chr5 | 7179967 | 9003079 | chr10 | 14017511 | 16804461 | 24 |
| chr5 | 9077498 | 9671485 | chr10 | 10483142 | 11973855 | 12 |
| chr5 | 9906161 | 10697434 | chr10 | 18314469 | 19688962 | 13 |
| chr5 | 11356680 | 11503072 | chr7 | 3284930 | 2967237 | 7 |
| chr5 | 11521825 | 11801891 | chr10 | 21054257 | 21341259 | 10 |
| chr5 | 11835994 | 12388460 | chr2 | 8977003 | 10200353 | 14 |
| chr5 | 12429963 | 12833086 | chr10 | 20800790 | 19780499 | 7 |
| chr5 | 13672035 | 14631195 | chr9 | 17948710 | 16992519 | 49 |
| chr5 | 14684040 | 15068100 | chr9 | 15212271 | 14781760 | 10 |
| chr5 | 15110227 | 15681458 | chr9 | 14056950 | 14646450 | 11 |
| chr5 | 15831631 | 16684368 | chr9 | 15352299 | 15795083 | 18 |
| chr5 | 16975806 | 19200820 | chr9 | 9360751 | 14496580 | 62 |
| chr5 | 19262921 | 20012310 | chr9 | 16879094 | 15876251 | 34 |
| chr5 | 20033129 | 22373833 | chr9 | 9389807 | 3465191 | 156 |
| chr5 | 22400034 | 22786296 | chr10 | 24534204 | 23839405 | 10 |
| chr5 | 22952897 | 24099252 | chr9 | 18019698 | 19732090 | 69 |
| chr5 | 23055267 | 23274124 | chr9 | 18486412 | 18313499 | 9 |
| chr5 | 23129243 | 23306727 | chr9 | 19201332 | 18356060 | 9 |
| chr5 | 24128704 | 29124459 | chr10 | 5136422 | 14913 | 567 |
| chr5 | 25115154 | 25151015 | chr10 | 3983518 | 4020332 | 8 |
| chr5 | 29540204 | 31899257 | chr9 | 3433690 | 6801 | 236 |
| chr5 | 30770144 | 30878507 | chr9 | 1316421 | 1437853 | 16 |
| chr6 | 25281 | 714471 | chr11 | 28070102 | 28784244 | 73 |
| chr6 | 729426 | 1415499 | chr3 | 1894209 | 3724479 | 79 |
| chr6 | 1421845 | 1668147 | chr3 | 19939402 | 19078654 | 29 |
| chr6 | 1675275 | 2298110 | chr3 | 16247883 | 18934007 | 33 |
| chr6 | 2103206 | 2294702 | chr3 | 18873678 | 17950905 | 7 |
| chr6 | 2306520 | 3534894 | chr3 | 16149842 | 10144898 | 55 |
| chr6 | 3557946 | 4260221 | chr3 | 21432643 | 19978379 | 66 |
| chr6 | 4275233 | 4605833 | chr3 | 3769116 | 4430924 | 19 |
| chr6 | 4669439 | 4924854 | chr11 | 26910530 | 27183038 | 28 |
| chr6 | 4938500 | 6796951 | chr11 | 31425741 | 29530124 | 198 |
| chr6 | 6810066 | 7036376 | chr11 | 29288455 | 29533538 | 22 |
| chr6 | 7058837 | 7482700 | chr11 | 29283943 | 28797573 | 41 |
| chr6 | 7507656 | 8747009 | chr11 | 12770307 | 17033803 | 60 |
| chr6 | 8452202 | 8613728 | chr11 | 16581060 | 15808304 | 11 |
| chr6 | 8690314 | 9184498 | chr11 | 19408636 | 17911028 | 26 |
| chr6 | 9230937 | 10023959 | chr11 | 21106053 | 22953439 | 55 |
| chr6 | 10034670 | 10994676 | chr11 | 25810538 | 26771987 | 83 |
| chr6 | 10258831 | 10366667 | chr0 | 844105 | 676190 | 15 |
| chr6 | 11091816 | 11835763 | chr11 | 24222445 | 23072268 | 33 |
| chr6 | 11867835 | 11932268 | chr11 | 24953256 | 25077945 | 6 |
| chr6 | 11935922 | 12039614 | chr0 | 385540 | 132289 | 11 |
| chr6 | 12107409 | 12742756 | chr11 | 20804160 | 19624768 | 61 |
| chr6 | 12754947 | 12923716 | chr11 | 27389927 | 27192232 | 19 |
| chr6 | 13123125 | 13206992 | chr11 | 26864689 | 26780117 | 6 |
| chr6 | 13721745 | 13801832 | chr11 | 24896219 | 24806323 | 7 |
| chr6 | 13816317 | 13986064 | chr11 | 20928262 | 21086377 | 7 |
| chr6 | 14516459 | 14750281 | chr11 | 25477529 | 25805445 | 24 |
| chr6 | 14773617 | 15030201 | chr11 | 25469842 | 25179061 | 18 |
| chr6 | 15047650 | 15208872 | chr11 | 19461130 | 19622042 | 16 |
| chr6 | 15299826 | 15901140 | chr11 | 27878756 | 27452795 | 40 |
| chr6 | 16734772 | 16877963 | chr11 | 28030338 | 27898107 | 13 |
| chr6 | 16966460 | 17581258 | chr3 | 5242260 | 6681258 | 27 |
| chr6 | 17600073 | 18742647 | chr3 | 22851205 | 24010309 | 104 |
| chr6 | 18763772 | 19645005 | chr3 | 6796691 | 9275468 | 35 |
| chr6 | 19490984 | 19639782 | chr3 | 9653364 | 9300307 | 7 |
| chr6 | 19673141 | 19868057 | chr3 | 24221537 | 24022778 | 16 |
| chr6 | 19891028 | 20515139 | chr3 | 22845260 | 22102090 | 57 |
| chr6 | 20543872 | 20723392 | chr5 | 3693795 | 3442287 | 10 |
| chr6 | 20752420 | 21281358 | chr5 | 2664789 | 3396744 | 49 |
| chr6 | 21296214 | 22609153 | chr5 | 2536303 | 1118194 | 128 |
| chr6 | 22617915 | 22706936 | chr5 | 4783053 | 4888072 | 17 |
| chr6 | 22722825 | 22860257 | chr8 | 29530241 | 29375439 | 14 |
| chr6 | 22894839 | 23953732 | chr8 | 9177194 | 7822808 | 113 |
| chr6 | 24172620 | 24645138 | chr8 | 7337277 | 6765372 | 39 |
| chr6 | 24653894 | 28360195 | chr8 | 2865747 | 6754971 | 477 |
| chr6 | 28362138 | 31068108 | chr8 | 2859316 | 18884 | 365 |
| chr7 | 80017 | 1597249 | chr1 | 17780907 | 14748789 | 130 |
| chr7 | 1601778 | 1772998 | chr1 | 27345583 | 27104679 | 20 |
| chr7 | 1778648 | 5813220 | chr1 | 4537278 | 29023 | 403 |
| chr7 | 3563889 | 3616739 | chr1 | 2166273 | 2226865 | 6 |
| chr7 | 6044837 | 6383159 | chr1 | 10777099 | 11129973 | 9 |
| chr7 | 7827285 | 8663744 | chr1 | 10591914 | 9916087 | 19 |
| chr7 | 8714694 | 9640431 | chr1 | 11411276 | 12219455 | 10 |
| chr7 | 9169521 | 9594454 | chr1 | 12069065 | 11769795 | 8 |
| chr7 | 9668277 | 10856810 | chr1 | 14542747 | 12274088 | 49 |
| chr7 | 10997778 | 12696763 | chr1 | 22040062 | 17849423 | 88 |
| chr7 | 12726259 | 12866013 | chr1 | 9306376 | 9678622 | 8 |
| chr7 | 12892618 | 12981868 | chr1 | 11362264 | 11227711 | 9 |
| chr7 | 13078474 | 13252443 | chr1 | 7287247 | 6996534 | 10 |
| chr7 | 13187120 | 14162842 | chr1 | 7133596 | 9264534 | 26 |
| chr7 | 14185100 | 14319392 | chr1 | 6345127 | 6653184 | 9 |
| chr7 | 14418174 | 15121996 | chr1 | 6179769 | 4678226 | 25 |
| chr7 | 15152099 | 15288841 | chr1 | 24094236 | 23188514 | 6 |
| chr7 | 15190509 | 16402962 | chr1 | 23953098 | 27045354 | 44 |
| chr7 | 16427193 | 16738211 | chr1 | 22395204 | 22093236 | 10 |
| chr7 | 16445483 | 16694966 | chr1 | 22367847 | 22989428 | 11 |
| chr7 | 16757203 | 17780657 | chr1 | 27448769 | 29641397 | 61 |
| chr7 | 17077539 | 17223465 | chr1 | 28466027 | 28161328 | 14 |
| chr7 | 17812412 | 18144899 | chr1 | 30230274 | 29712776 | 38 |
| chr7 | 17960221 | 18202556 | chr1 | 30060634 | 30326658 | 7 |
| chr7 | 18213477 | 19099090 | chr1 | 31627747 | 30340751 | 108 |
| chr7 | 19119771 | 22434049 | chr1 | 31674796 | 35356300 | 379 |
| scaffold72 | 8838 | 168556 | chr2 | 1637086 | 1836799 | 6 |
| scaffold78 | 7240 | 130492 | chr7 | 22995631 | 23111882 | 7 |

**Table S10. Positively selected genes in *Cucumis hystrix* with “response to biotic stimulus” (GO:0009607).**

| Gene ID | Homolog in Arabidopsis | Description (IPR) | FDR | Number of species | Number of PSS^a^ |
| --- | --- | --- | --- | --- | --- |
| Chy11G190090.1 | AT3G50440 | Alpha/beta hydrolase fold-1 | 3.36E-02 | 5 | 1 |
| Chy1G001840.1 | AT5G09650 | Inorganic pyrophosphatase | 2.13E-02 | 7 | 1 |
| Chy1G011120.1 | AT1G71930 | NAC domain | 3.36E-02 | 4 | 3 |
| Chy2G032350.1 | AT3G21630 | Protein kinase domain | 4.98E-02 | 6 | 8 |
| Chy2G044550.1 | AT5G57685 | - | 1.21E-02 | 7 | 3 |
| Chy2G044620.1 | AT4G25780 | Cysteine-rich secretory protein | 2.27E-02 | 7 | 2 |
| Chy3G060900.1 | AT2G45180 | Hydrophobic seed protein | 4.59E-05 | 6 | 2 |
| Chy4G068910.1 | AT1G51580 | K Homology domain | 1.61E-03 | 7 | 5 |
| Chy4G076630.1 | AT1G59870 | ABC transporter-like | 5.92E-04 | 7 | 4 |
| Chy7G137680.1 | AT4G35580 | NAC domain | 2.65E-02 | 7 | 18 |
| Chy9G171820.1 | AT5G24660 | - | 3.30E-02 | 6 | 1 |
| ChyUNG234630.1 | AT5G06720 | Plant peroxidase | 7.31E-08 | 5 | 34 |

^a^PSS: positively selected sites.

**Table S11. Primers used to conduct PCR to verify the specificity of *Cucumis hystrix* chromosome-specific markers**

| Primer ID | Forward Primer (5’-3’) | Reverse Primer (5’-3’) |
| --- | --- | --- |
| 1-1 | ATATGAGTAGAAAGCAGATGAC | AGAGAAGAGACACAAAAGTTG |
| 1-2 | AGCCTTTTTCCTACACCATAA | ATTCCCTAGATCCCTTGACTG |
| 1-3 | CGATTTCTTCACTTCCTCTTT | CTGGACACTAACTATGATTTTC |
| 2-1 | AGGTGTTCGTTGTAGGTCTTA | CAAAATAGGTTGATGTCTCGTT |
| 2-2 | AGTTAGGGTGTGAATTGGCTT | GCATCAAAGAATGTGTGGGTT |
| 2-3 | GTCTTTTACTCTTGGTTTTCG | TAATCGTTCCACCCATCACTA |
| 3-1 | AATAAGCTCACATGTGTCGAT | ATACTACTTGCACCTCCTACG |
| 3-2 | CAATAGAATCCATATCTTTGA | GTCATTCGTTTACTATCACAT |
| 3-3 | TTCAAGTGCAGTATAGGGTGG | TTAGAAGGACAAATGTAGCGG |
| 4-1 | AACGCTACTGTTGTTGACCCT | GAGAAATGAAAAGCATGGAGAT |
| 4-2 | GTGTGGATACCAAATCAACTA | CGTTTTTACAAGTACACGATC |
| 4-3 | ATCTATAGGAACGTACAAGGT | ATATCCAAGAAAGGGGACAAC |
| 5-1 | CCTATTCTTCTCACGCGGTAT | TTTCTTTGGTGTGTGGTTGTC |
| 5-2 | ACCAGTGATTGACTTTTATCG | ATATGGAACATTGTATCCCAC |
| 5-3 | GAATAGGGAAAAGTTGCCGTT | TGATGCTGTCAAGTCGAGTCA |
| 6-1 | GAAGAAAATGGAAGGTGTAGA | CTCTTTCGTGAAAATACTTGAG |
| 6-2 | GTAAGAGGTGACGATGTTTCC | CTTAATCCTAATTTCTTGGACG |
| 6-3 | CGACAAGTTTGACGTTGATAT | CTAGTTTGAACTGCGTGTATTA |
| 7-1 | TATACCCAACCAACCGATTCC | ACTTTGAGGGCTTGTGTCTCC |
| 7-2 | CCGTCATCCTTACAAACGTCA | ACAACCCACCCTTGGAAAATA |
| 7-3 | ATGTTGAATGACCCTTTGTTAG | GATTTTGTGAACGGTTTATGAG |
| 8-1 | ATCGTAGAAAGGAAAGGAGAC | GATCAATTGTTGGGAGTGACT |
| 8-2 | AATGTTGACTTTGAACCTCCT | TTGCTCACTATTTTCTCCTAC |
| 8-3 | TCGTTTTACGTGTTACTCGAT | GTGGAATTAGAACTTTTGGCTT |
| 9-1 | CCATCACCACTAACACACTTT | TCTACCGCCTACTCTCATCAC |
| 9-2 | GCAGAAACCTGACAATTCCCT | CGTATAACACCTTCCCGCTAA |
| 9-3 | AGAAAATTTGGATGAAAACTAGC | ATAGAGAAAACGAACACGACT |
| 10-1 | AAGAGCCTATAAGAACCCACA | ACAAAGAAGACTCAACCCAAC |
| 10-2 | TACATTCCAATAGGGTGCCGT | GCCGTACTCACAGATTAAAAAA |
| 10-3 | TGAATCACTTGTCCAACATAC | CTCCTTTAATGATTTCATCAAC |
| 11-1 | CTTACGCTTTTGGGATAGTCT | CATAGGCATCTACAGTTTCAAT |
| 11-2 | ACTACACTAGCGAGCAAGGGC | ACAGTAATGGTTTGGAAACGG |
| 11-3 | GTAATATTTTCCATCAAAACTGC | CAAACAACCATAATTTAGTCGTA |
| 12-1 | AGAAATAGCCCTCAACAAAGA | CCAACCAATTATATCCTCACAT |
| 12-2 | GGACCCAACCTTACACCCTTA | TTCGAGTTTCAATTCCTCACC |
| 12-3 | AGAAGTCTGGCATGTGTGTGA | AGAAGGTTTGTTGGAGTTTGG |
